# LotuS2: an ultrafast and highly accurate tool for amplicon sequencing analysis

**DOI:** 10.1186/s40168-022-01365-1

**Published:** 2022-10-19

**Authors:** Ezgi Özkurt, Joachim Fritscher, Nicola Soranzo, Duncan Y. K. Ng, Robert P. Davey, Mohammad Bahram, Falk Hildebrand

**Affiliations:** 1 Gut Microbes & Health, Quadram Institute Bioscience, Norwich Research Park, Norwich, Norfolk, NR4 7UQ UK; 2 Earlham Institute, Norwich Research Park, Norwich, Norfolk, NR4 7UZ UK; 3 Department of Ecology, Swedish University of Agricultural Sciences, Ulls väg 16, 756 51 Uppsala, Sweden; 4 Institute of Ecology and Earth Sciences, University of Tartu, Lai St, 40 Tartu, Estonia

**Keywords:** Microbiome, Short read, Long read, Amplicon sequencing, Amplicon data analysis, 16S rRNA, ITS

## Abstract

**Background:**

Amplicon sequencing is an established and cost-efficient method for profiling microbiomes. However, many available tools to process this data require both bioinformatics skills and high computational power to process big datasets. Furthermore, there are only few tools that allow for long read amplicon data analysis. To bridge this gap, we developed the LotuS2 (less OTU scripts 2) pipeline, enabling user-friendly, resource friendly, and versatile analysis of raw amplicon sequences.

**Results:**

In LotuS2, six different sequence clustering algorithms as well as extensive pre- and post-processing options allow for flexible data analysis by both experts, where parameters can be fully adjusted, and novices, where defaults are provided for different scenarios.

We benchmarked three independent gut and soil datasets, where LotuS2 was on average 29 times faster compared to other pipelines, yet could better reproduce the alpha- and beta-diversity of technical replicate samples. Further benchmarking a mock community with known taxon composition showed that, compared to the other pipelines, LotuS2 recovered a higher fraction of correctly identified taxa and a higher fraction of reads assigned to true taxa (48% and 57% at species; 83% and 98% at genus level, respectively). At ASV/OTU level, precision and F-score were highest for LotuS2, as was the fraction of correctly reported 16S sequences.

**Conclusion:**

LotuS2 is a lightweight and user-friendly pipeline that is fast, precise, and streamlined, using extensive pre- and post-ASV/OTU clustering steps to further increase data quality. High data usage rates and reliability enable high-throughput microbiome analysis in minutes.

**Availability:**

LotuS2 is available from GitHub, conda, or via a Galaxy web interface, documented at http://lotus2.earlham.ac.uk/.

Video Abstract

**Supplementary Information:**

The online version contains supplementary material available at 10.1186/s40168-022-01365-1.

## Background

The field of microbiome research has been revolutionized in the last decade, owing to methodological advances in DNA-based microbial identification. Amplicon sequencing (also known as metabarcoding) is one of the most commonly used techniques to profile microbial communities based on targeting and amplifying phylogenetically conserved genomic regions such as the 16S/18S ribosomal RNA (rRNA) or internal transcribed spacers (ITS) for identification of bacteria and eukaryotes (especially fungi), respectively [1, 2]. The popularity of amplicon sequencing has been growing due to its broad applicability, ease-of-use, cost-efficiency, streamlined analysis workflows as well as specialist applications such as low biomass sampling [3].

Alas, amplicon sequencing comes with several technical challenges. These include primer biases [4], chimeras occurring in PCR amplifications [5], rDNA copy number variations [6], and sequencing errors that frequently inflate observed diversity [7]. Although modern read error corrections can already significantly decrease artifacts of sequencing errors [8], some of the biases can be further corrected in the pre- and post-processing of reads and OTUs/ASVs, respectively. To process amplicon sequencing data from raw reads to taxon abundance tables, several pipelines have been developed, such as mothur [9], QIIME 2 [10], DADA2 [8], PipeCraft 2 [11], and LotuS [12]. These pipelines differ in their data processing and sequence clustering strategies, reflected in differing execution speed and resulting amplicon interpretations [12, 13].

Here, we introduce Lotus2, designed to improve reproducibility, accuracy, and ease of amplicon sequencing analysis. LotuS2 offers a completely refactored installation, including a web interface that is freely deployable on Galaxy clusters. During development, we focused on all steps of amplicon data analysis, including processing raw reads to abundance tables as well as improving taxonomic assignments and phylogenies of operational taxonomic units (OTUs [14]; or amplicon sequence variants (ASVs [15];) at the highest quality with the latest strategies available.

Pre- and post-processing steps were further improved compared to the predecessor “LotuS1”: the read filtering program sdm (simple demultiplexer) and the taxonomy inference program LCA (least common ancestor) were refactored and parallelized in C++. LotuS2 uses a ‘seed extension’ algorithm that improves the quality and length of OTU/ASV representative DNA sequences. We integrated numerous features such as additional sequence clustering options (DADA2, UNOISE3, VSEARCH and CD-HIT), advanced read quality filters based on probabilistic and Poisson binomial filtering, and curated ASVs/OTUs diversity and abundances (LULU, UNCROSS2, ITSx, and host DNA filters). LotuS2 can also be integrated in complete workflows. For instance, the microbiome visualization-centric pipeline CoMA [16] uses LotuS1/2 at its core to estimate taxon abundances.

Here, we evaluated LotuS2 in reproducing microbiota profiles in comparison to contemporary amplicon sequencing pipelines. Using three independent datasets, we found that LotuS2 consistently reproduces microbiota profiles more accurately and reconstructs a mock community with the highest overall precision.

## Materials and methods

### Design philosophy of LotuS2

**Fig. 1:**
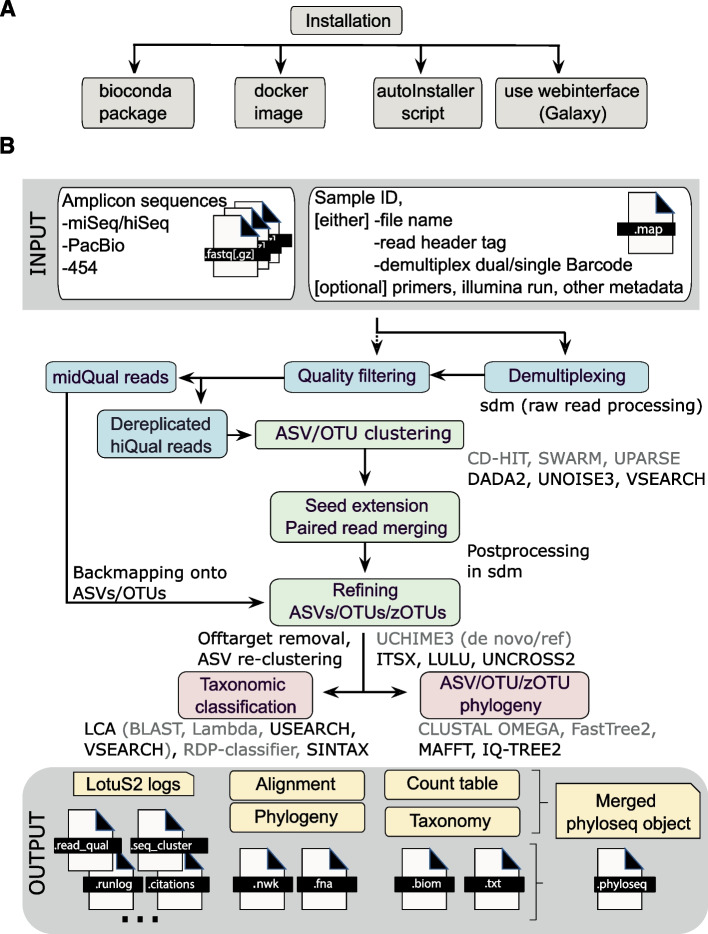
Workflow of the LotuS2 pipeline. **A** LotuS2 can be installed either through (i) Bioconda, (ii) GitHub with the provided autoInstaller script, or (iii) using a Docker image. Alternatively, (iv) Galaxy web servers can also run LotuS2 (e.g., https://usegalaxy.eu/). **B** LotuS2 accepts amplicon reads from different sequencing platforms, along with a map file that describes barcodes, file locations, sample IDs, and other information. After demultiplexing and quality filtering, high-quality reads are clustered into either ASVs or OTUs. The optimal sequence representing each OTU/ASV is calculated in the seed extension step, where read pairs are also merged. Mid-quality reads are subsequently mapped onto these sequence clusters to increase cluster representation in abundance matrices. From OTU/ASV sequences, a phylogenetic tree is constructed, and each cluster is taxonomically assigned. These results are made available in multiple standard formats, such as tab-delimited files, .biom, or phyloseq objects to enable downstream analysis. New options in LotuS2 for each step are denoted with black colour whereas options in grey font were already available in LotuS

Overestimating observed diversity is one of the central problems in amplicon sequencing, mainly due to sequencing errors [7, 17]. The second read pair from Illumina paired-end sequencing is generally lower in quality [18] and can contain more errors than predicted from Phred quality scores alone [19, 20]. Additionally, merging reads can introduce chimeras due to read pair mismatches [21]. The accumulation of errors over millions of read pairs can impact observed biodiversity, so essentially is a multiple testing problem. To avoid overestimating biodiversity, LotuS2 uses a relatively strict read filtering during the error-sensitive sequence clustering step. This is based on (i) 21 quality filtering metrics (e.g., average quality, homonucleotide repeats, and removal of reads without amplicon primers), (ii) probabilistic and Poisson binomial read filtering [18, 22], (iii) filtering reads that cannot be dereplicated (clustered at 100% nucleotide identity) either within or between samples, and (iv) using only the first read pair from paired-end Illumina sequencing platforms. These reads are termed “high-quality” reads in the pipeline description and are clustered into OTUs/ASVs, using one of the sequence clustering programs (Fig. 1B).

However, filtered out “mid-quality” sequences are partly recovered later in the pipeline, during the seed extension step. LotuS2 will reintroduce reads failing dereplication thresholds or being of “mid-quality” by mapping these reads back onto high-quality OTUs/ASVs if matching at ≥ 97% sequence identity. In the “seed extension” step, the optimal sequence representing each OTU/ASV is determined by comparing all (raw) reads clustered into each OTU/ASV. The best read (pair) is then selected based on the highest overall similarity to the consensus OTU/ASV, quality, and length, which can then be merged in case of paired read data. Thereby, the seed extension step enables more reads to be included in taxon abundance estimates, as well as enabling longer ASV/OTU representative sequences to be used during taxonomic classifications and the reconstruction of a phylogenetic tree.

### Implementation of LotuS2

#### Installation

LotuS2 can be accessed either through major software repositories such as (i) Bioconda, (ii) as a Docker image, or (iii) GitHub (accessible through http://lotus2.earlham.ac.uk/) (Fig. 1A). The GitHub version comes with an installer script that downloads the required databases and installs and configures LotuS2 with its dependencies. Alternatively, we provide iv) a wrapper for Galaxy [23] allowing installation of LotuS2 on any Galaxy server from the Galaxy ToolShed. LotuS2 is already available to use for free on the UseGalaxy.eu server (https://usegalaxy.eu/), where raw reads can be uploaded and analysed (Supplementary Figure S1). While LotuS2 is natively programmed for Unix (Linux, macOS) systems, other operating systems are supported through the Docker image or the Galaxy web interface.

#### Input

LotuS2 is designed to run with a single command, where the only essential flags are the path to input files (fastq(.gz), fna(.gz) format), output directory, and mapping file. The mapping file contains information on sample identifiers, demultiplexing barcodes, or file paths to already demultiplexed files and can be either automatically generated or provided by the user. The sequence input is flexible, allowing simultaneous demultiplexing of read files and/or integration of already demultiplexed reads.

LotuS2 is highly configurable, enabling user-specific needs beyond the well-defined defaults. There are 63 flags that can be user-modified, including dereplication filtering thresholds (-derepMin), sequencing platform (-p), amplicon region (-amplicon_type), or OTU/ASV post-processing (e.g., -LULU option to remove erroneous OTUs/ASVs [24]). In addition, read filtering criteria can be controlled through 32 detailed options via custom config files (defaults are provided for Illumina MiSeq, hiSeq, novaSeq, Roche 454, and PacBio HiFi).

#### Output

The primary output is a set of tab-delimited OTU/ASV count tables, the phylogeny of OTUs/ASVs, their taxonomic assignments, and corresponding abundance tables at different taxonomic levels. These are summarized in .biom [25] and phyloseq objects [26], that can be loaded directly by other software, such as R and Python programming languages, for downstream analysis.

Furthermore, a detailed report of each processing step can be found in the log files which contain commands of all used programs (including citations and versions) with relevant statistics. We support and encourage users to conduct further analysis in statistical programming languages such as R, Python, or MATLAB and using analysis packages such as phyloseq [26], documented in tutorials at http://lotus2.earlham.ac.uk/.

#### Pipeline workflow

Most of LotuS2 is implemented in Perl 5.1; computational or memory intensive components like simple demultiplexer (sdm) and LCA (least common ancestor) are implemented in C++ (see Fig. 1B for pipeline workflow). Demultiplexing, quality filtering, and dereplication of reads is implemented in sdm. Taxonomic post-processing is implemented in LCA. Six sequence clustering methods are available: UPARSE [18], UNOISE3 [27], CD-HIT [28], SWARM [29], DADA2 [8], and VSEARCH [30].

In the “seed extension” step, a unique representative read of a sequence cluster is chosen, based on quality and merging statistics. Each sequence cluster, termed ASVs in the case of DADA2, OTUs otherwise^1^, is represented by a high confidence DNA sequence (see Design Philosophy of LotuS2 for more information).

OTUs/ASVs are further post-processed to remove chimeras, either *de novo* and/or reference based using the program UCHIME3 [31] or VSEARCH-UCHIME [30]. By default, ITS sequences are extracted using ITSx [32]. Highly resolved OTUs/ASVs are then curated based on sequence similarity and co-occurrence patterns using LULU [24]. False-positive OTU/ASV counts can be filtered using the UNCROSS2 algorithm [33]. OTUs/ASVs are by default aligned against the phiX genome, a synthetic genome often included in Illumina sequencing runs, using Minimap2 [34]; and OTUs/ASVs that produce significant matches against the phiX genome are subsequently removed. Additionally, the user can filter for host contamination by providing custom genomes (e.g., human reference), as host genome reads are often misclassified as bacterial 16S by existing pipelines [3].

Each OTU/ASV is taxonomically classified using one of RDP classifier [35], SINTAX [36], or by alignments to reference database(s), using the custom “LCA” (least common ancestor) C++ program. Alignments of OTUs/ASVs with either Lambda [37], BLAST [38], VSEARCH [30], or USEARCH [39] are compared against a user-defined range of reference databases. These databases cover the 16S, 18S, 23S, 28S rRNA genes, and the ITS region; by default, a Lambda alignment against the SILVA database is used [40]. Other databases bundled with LotuS2 include Greengenes [41], HITdb [42], PR2 [43], beetax (bee gut-specific taxonomic annotation) [44], and UNITE (fungal ITS database) [45]. In addition, users can provide reference databases (a fasta file and a tab-delimited taxonomy file, see "–refdb" flag documentation in the LotuS2 help). These databases can be used by themselves or in conjunction with the bundled ones. From mappings against one or several reference databases, the least common ancestor for each OTU/ASV is calculated using LCA. Priority is given to deeply resolved taxonomies, sorted by the earlier listed reference databases. LotuS2 can also be used to analyse amplicons from other phylogenetically conserved genomic regions (e.g., Cytochrome c oxidase subunit I (COI) or dissimilatory sulfite reductase (dsr)). For these cases, users have to provide custom reference databases and taxonomic assignments (via -refdb flag, see above). For inferring phylogenetic trees, multiple sequence alignments for all OTUs/ASVs are calculated with either MAFFT [46] or Clustal Ω [47]; from these a maximum likelihood phylogeny is constructed using either fasttree2 [48] or IQ-TREE 2 [49]. User discretion is advised, as ITS amplicons might be less suitable for inferring reliable phylogenies.

If the pipeline should fail at any step, a comprehensive error report with suggestions for follow up steps is automatically provided to the user; bugs should be submitted to “https://github.com/hildebra/lotus2”.

### Benchmarking amplicon sequencing pipelines

To benchmark the computational performance and reproducibility, we compared LotuS2’s performance to commonly used amplicon sequencing pipelines including mothur [9], PipeCraft 2 [11], DADA2 [8], and QIIME 2 [10]. We relied, where possible, on default options or standard operating procedure (SOPs) provided by the respective developers (mothur: https://mothur.org/wiki/miseq_sop/;

PipeCraft 2: https://pipecraft2-manual.readthedocs.io/en/stable/user_guide.html; QIIME 2: https://docs.qiime2.org/2021.11/tutorials/moving-pictures/, and DADA2: https://benjjneb.github.io/dada2/tutorial.html). We benchmarked PipeCraft 2 using the demultiplexed raw reads from LotuS2. DADA2 cannot demultiplex raw reads and in these cases, LotuS2 demultiplexed raw reads were also used as DADA2 input.

Our benchmarking scripts are available at https://github.com/ozkurt/lotus2_benchmarking (see Supplementary information). Several sequence cluster algorithms were benchmarked, for LotuS2: DADA2 [8], UPARSE [18], UNOISE3 [27], CD-HIT [28], and VSEARCH [30]; for QIIME 2: DADA2 and Deblur [50]; DADA2 supporting natively only DADA2 clustering; for mothur: OptiClust; for PipeCraft 2: VSEARCH, and for LotuS1: UPARSE. For taxonomic classification, SILVA138.1 [40] was used in all pipelines.

ITS amplicons were clustered with CD-HIT, UPARSE, and VSEARCH and filtered by default using ITSx [32] in LotuS2. ITSx identifies likely ITS1, 5.8S, and ITS2 and full-length ITS sequences, and sequences not within the confidence interval are discarded in LotuS2. In analogy, QIIME 2-DADA2 uses q2-ITSxpress [51] that also removes unlikely ITS sequences.

Error profiles during ASV clustering were inferred separately for the samples sequenced in different MiSeq runs during DADA2 and Deblur clustering in all pipelines. We truncated the reads into the same length (200 bases, default by LotuS2) in all pipelines while analysing the datasets. Primers were removed from the reads, where supported by the pipeline in question.

### Measuring computational performance of amplicon sequencing pipelines

When benchmarking pipelines, processing steps were separated into 5 categories in each tested pipeline: (a) Pre-processing (demultiplexing if required, read filtering, primer removal, and read merging for QIIME 2-Deblur), (b) sequence clustering (clustering + refining of the clusters and denoising for QIIME 2-DADA2, (c) OTU/ASV taxonomic assignment, (d) construction of a phylogenetic tree (the option is available only in mothur, QIIME 2, and LotuS2 and applied only for the 16S datasets), and (e) removal of host genome (the option is available only in QIIME 2 and LotuS2). In mothur, sequence clustering and taxonomic assignment times were added since these pipeline commands are entangled (https://mothur.org/wiki/miseq_sop/).

### Data used in benchmarking pipeline performance

Four datasets with different sample characteristics (with respect to, e.g., compositional complexity, target marker and region, and amplicon length) were analyzed: (i) *Gut-16S* dataset [12]: 16S rRNA gene amplicon sequencing of 40 human faecal samples in technical replicates that were sequenced in separate MiSeq runs, totalling 35,412,313 paired-end reads. Technical replicates were created by extracting DNA twice from each faecal sample. Primer sequences were not available for this dataset [12]. Since the Illumina runs were not demultiplexed, pipelines had to demultiplex these sequences, as applicable (please see the Computational performance and data usage section for further details). (ii) *Soil-16S* dataset: 16S rRNA gene amplicon sequencing of two technical replicates (a single DNA extraction per sample) from 50 soil samples, that were sequenced in separate MiSeq runs, totalling 11,820,327 paired-end reads. PCR reactions were conducted using the 16S rRNA region primers 515F (GTGYCAGCMGCCGCGGTAA) and 926R (GGCCGYCAATTYMTTTRAGTTT). The soil-16S dataset was already demultiplexed, requiring pipelines to work with paired FASTQ files per sample. (iii) *Soil-ITS* dataset: ITS amplicon sequencing of 50 technical replicates of soil samples (a single DNA extraction per sample), sequenced in two independent Illumina MiSeq runs, totalling 6,006,089 paired-end reads. The ITS region primers gITS7ngs_201 (GGGTGARTCATCRARTYTTTG) and ITS4ngsUni_201 (CCTSCSCTTANTDATATGC) [52] were used to amplify DNA extracted from soil samples. The soil-ITS dataset was already demultiplexed.

(iv) *Mock* dataset [53]: This was a microbial mock community with known species composition, *mock-16* [53]. The mock dataset comprised a total of 59 strains of Bacteria and Archaea, representing 35 bacterial and 8 archaeal genera. The mock community was sequenced on an Illumina MiSeq (paired-end) by targeting the V4 region of the 16S rRNA gene using the primers 515F (GTGCCAGCMGCCGCGGTAA) and 806R (GGACTACHVGGGTWTCTAAT) [53]. This dataset was demultiplexed and contained 593,868 paired reads.

### Benchmarking the computational performance of amplicon sequencing pipelines

To evaluate the computational performance of LotuS2 in comparison to mothur, QIIME 2 [10], DADA2 [8], and the last released version of LotuS [12] (v1.62 from Jan 2020; called LotuS1 here), all pipelines were run with 12 threads on a single computer free of other workloads (CPU: Intel(R) Xeon(R) Gold 6130 CPU @ 2.10 GHz, 32 cores, 375 GB RAM). To reduce the influence of network latencies on pipeline execution, all temporary, input, and output data were stored on a local SSD hard drive. PipeCraft 2 is not designed for high performance computing cluster execution (https://pipecraft2-manual.readthedocs.io/en/stable/installation.html#windows) and was therefore excluded from computational performance benchmarking; however, the gut-16S and soil-16S datasets using default options and 6 cores where possible was executed in a laptop in > 8 h (excluding the demultiplexing step) and in > 24 h, respectively.

The remaining pipelines were run three times consecutively to account for pre-cached data and to obtain average execution time and maximum memory usage. To calculate the fold differences in execution speed between pipelines, the average time of QIIME 2, mothur, and DADA2 to complete the analysis was divided by the average time by all LotuS2 runs (using different clustering options). The average of these numbers across the gut-16S, soil-16S, and soil-ITS datasets was used to estimate the average speed advantage of LotuS2.

### Benchmarking reproducibility of amplicon sequencing pipelines

Technical replicates of the soil and gut samples were used to estimate the reproducibility of the microbial community composition between replicates. This was measured by calculating beta and alpha diversity differences between technical replicate samples. To calculate beta diversity, either Jaccard (measuring presence/absence of OTUs/ASVs) or Bray-Curtis dissimilarity (measuring both presence/absence and abundances of OTUs/ASVs) were computed between technical replicate samples. Before computing Bray-Curtis distances, abundance matrices were normalized. Jaccard distances between samples were calculated by first rarefying abundance matrices to an equal number of reads (to the size of the first sample having > 1000 read counts) per sample using RTK [54]. Significance of pairwise comparisons of the pipelines in beta diversity differences was calculated using the ANOVA test where Tukey’s HSD (honest significant differences) test was used as a post hoc test in R.

To calculate alpha diversity, abundance data were first rarefied to an equal number of reads per sample. Significance of each pairwise comparison in alpha diversity was calculated based on a paired Wilcoxon test, pairing technical replicates.

### Analysis of the mock community

We used an already sequenced mock community [53] of known relative composition and with sequenced reference genomes available. Firstly, taxonomic abundance tables (taxonomic assignments based on SILVA 138.1 [40] in all pipelines) were compared to the expected taxonomic composition of the sequenced mock community*.* Precision was calculated as (TP/(TP + FP)), recall as (TP/(TP + FN)), and F-score as (2*precision*recall/(precision+recall)), TP (true positive) being taxa present in the mock and correctly identified as present, FN (false negative) being taxa present in the mock but not identified as present, and FP (false positive) being taxa absent in the mock but identified as present. The fraction of read counts assigned to true positive taxa was calculated based on the sum of the relative abundance of all true positive taxa. These scores were calculated at species and genus levels.

Secondly, we investigated the precision of reported 16S rRNA nucleotide sequences, representing each OTU or ASV, by calculating the nucleotide similarity between ASVs/OTUs and the known reference 16S rRNA sequences. To obtain the nucleotide similarity, we aligned ASV/OTU DNA sequences from tested pipelines via BLAST to a custom reference database that contained the 16S rRNA gene sequences from the mock community (https://github.com/caporaso-lab/mockrobiota/blob/master/data/mock-16/source/expected-sequences.fasta), using the –taxOnly option from LotuS2. The BLAST % nucleotide identity at > 50% horizontal OTU/ASV sequence coverage is subsequently used to calculate the best matching 16S rRNA sequence per ASV/OTU.

## Results

We analyzed four datasets to benchmark the computational performance and reliability of the pipelines. The datasets consisted either of technical replicates (gut-16S, soil-16S, and soil-ITS) or a mock community. Technical replicates were used to evaluate the reproducibility of community structures and were chosen to represent different biomes (gut and soil) using different 16S rRNA amplicon primers (gut-16S and soil-16S), or ITS sequences (soil-ITS) as well as a synthetic mock community of known composition.

### Computational performance and data usage

**Fig. 2:**
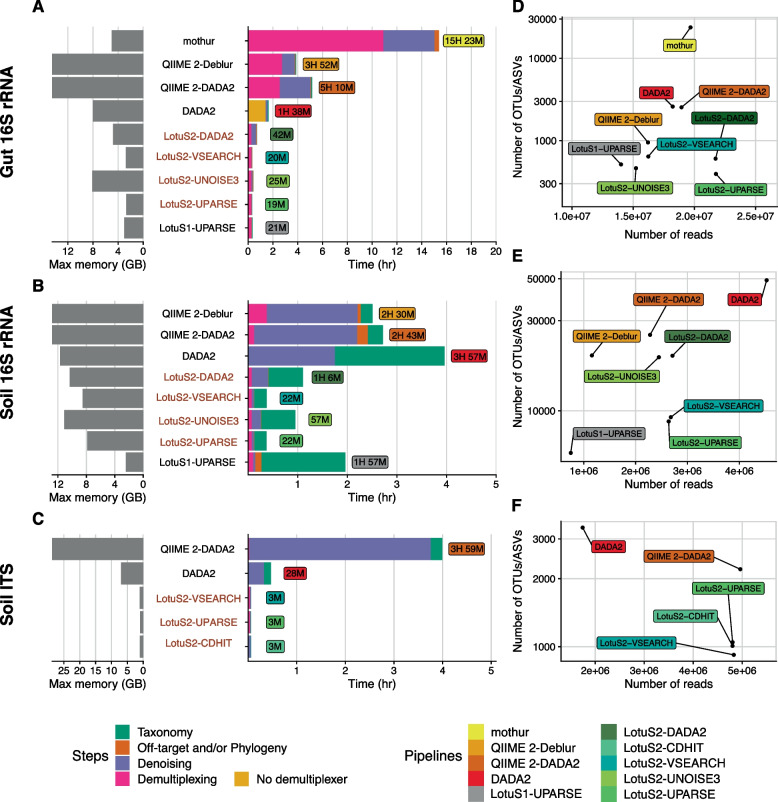
Computational performance of amplicon sequencing pipelines. 16S rRNA amplicon MiSeq data from **A** gut-16S, **B** soil-16S, and **C** soil-ITS samples were processed to benchmark resource usage of each pipeline, run on the same system under equal conditions (12 cores, max 150 Gb memory). In all pipelines, OTUs/ASVs were classified by similarity comparisons to SILVA 138.1. In LotuS2, Lambda was used to align sequences for all clustering algorithms. Pipeline runs were separated by common steps (pre-processing, sequence clustering, taxonomic classification, and phylogenetic tree construction and/or off-target removal). Because native DADA2 cannot demultiplex reads, we used the average demultiplexing time of QIIME 2 and LotuS2 (LotuS2 demultiplexed, unfiltered reads were provided to DADA2). Since phylogenetic trees based on ITS sequences may lead to erroneous phylogenies [55], we did not include the phylogenetic tree construction step in the analysis of the soil-ITS dataset. LotuS2 runs are labelled with red color. **D**, **E**, **F** Data usage efficiency of each tested pipeline, by comparing the number of sequence clusters (OTUs or ASVs) to retrieved read counts in the final output matrix of each pipeline. Note that mothur results for soil-16S are not shown, because the pipeline rejected all sequences at the default parameters

The complete analysis of the gut-16S dataset was fastest in LotuS2 (on average 35, 12, 9, and 3.8 times faster than mothur, QIIME 2-DADA2, QIIME 2-Deblur, and native DADA2, respectively, Fig. 2A). Note that since DADA2 could not demultiplex the dataset, the average of LotuS2 and QIIME2 demultiplexing times were used instead. LotuS2 was also faster in the analysis of the soil-16S dataset compared to the other tested pipelines (5.7, 3.5, and 3.5 times faster than DADA2, QIIME 2-DADA2, and QIIME 2-Deblur, respectively, Fig. 2B). The difference in speed between LotuS2 and QIIME 2 was more pronounced in the analysis of the soil-ITS dataset, where LotuS2 was on average 69 times faster than QIIME 2 and DADA2 (Fig. 2C).

LotuS2 also outperformed other pipelines in the case of the gut-16S dataset (on average LotuS2 was 15 times faster) compared to the soil dataset (average 4.2). This difference stems mainly from the demultiplexing step, where LotuS2 is significantly faster. The sequence clustering step was fastest using the UPARSE algorithm with an average 60-fold faster run time than sequence clustering in other pipelines. Averaged over these three datasets, LotuS2 was 29 times faster than other pipelines.

Taxonomic classification of OTUs/ASVs was also faster in LotuS2 (~ 5 times faster for gut-16S and 2 times for soil-16S). However, this strongly depends on the total number of OTUs/ASVs for all pipelines. For example, the default naïve-Bayes classifier [56] in QIIME 2 is faster than the LotuS2 taxonomic assignment in this benchmark (using Lambda LCA against the SILVA reference database). Nevertheless, LotuS2 also offers taxonomic classifications via RDP classifier [35] or SINTAX [36], both of which are significantly faster.

Compared to LotuS1, LotuS2 was on average 3.2 times faster, likely related to refactored C++ programs that can take advantage of multiple CPU threads (Fig. 2A, B). In its fastest configuration (using “UPARSE” option in clustering and “RDP” to assign taxonomy), the gut and soil 16S rRNA datasets can be processed with LotuS2 in under 20 min and 12 min, respectively, using < 10 GB of memory and 4 CPU cores.

Despite using similar clustering algorithms (e.g., DADA2 clustering is available in DADA2, QIIME 2, and LotuS2), the tested pipelines apply different pre- and post-processing algorithms to raw sequence reads and clustered ASVs and OTUs, leading to differing ASV/OTU numbers and retrieved reads (the total read count in the ASV/OTU abundance matrix) (Supplementary Table S1 and Fig. 2D–F). DADA2 typically estimated the highest number of ASVs, but the number of retrieved reads varied strongly between datasets. QIIME 2-DADA2 estimated fewer ASVs than DADA2, but more ASVs than LotuS2-DADA2, while mapping fewer reads than LotuS2. Although retrieving a smaller number of reads, QIIME 2-Deblur reported comparable numbers of ASVs to LotuS2, despite the differences in clustering algorithms. PipeCraft 2 using VSEARCH clustering retrieved slightly higher number of reads in the final output matrix than LotuS2-VSEARCH; but it also reported a considerably higher number of OTUs (Supplementary Figure S2). Although retrieving a smaller number of reads, QIIME 2-Deblur reported comparable numbers of ASVs to LotuS2, despite the differences in clustering algorithms. mothur performed differently in the gut-16S and soil-16S datasets, where it estimated either the highest number of OTUs or could not complete the analysis since all the reads had been filtered out, respectively. Overall, LotuS2 often reported the fewest ASVs/OTUs, while including more sequence reads in abundance tables. This indicates that LotuS2 has a more efficient usage of input data while covering a larger sequence space per ASV/OTU.

### Benchmarking the reproducibility of community compositions

**Fig. 3:**
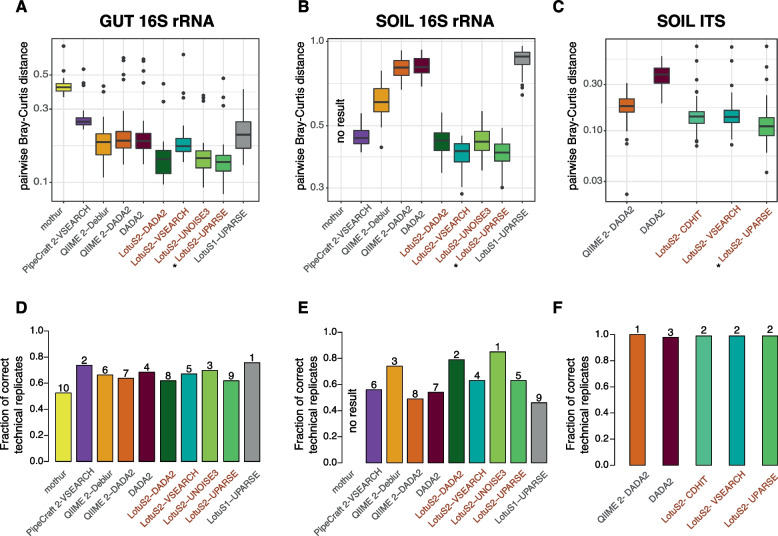
Reproducibility from different amplicons sequence data analysis pipelines. Three independent datasets were used to represent different biomes and amplicon technologies, using **A**, **D** human faecal samples (16S rRNA gene, *N* = 40 replicates). **B**, **E** soil samples (16S rRNA gene, *N* = 50 replicates), and **C**, **F** soil samples (ITS 2, *N* = 50 replicates). **A**–**C** Bray-Curtis distances among technical replicate samples were used to assess the reproducibility of community compositions by different pipelines. The pipeline with the lowest BCd in each subfigure is denoted with a star (*). The significance of pairwise comparisons of each pipeline was calculated using the Tukey’s HSD test (Supplementary Table S2). **D**–**F** Further, the fraction of technical replicates being closest to each other (BCd) was calculated to simulate identifying technical replicates without additional knowledge. Numbers above bars are the ordered pipelines performing best. Lower Bray-Curtis distances between technical replicates and a higher fraction of correct technical replicates indicate better reproducibility. LotuS2 runs are labelled with red color

Next, we assessed the reproducibility of community compositions between pipelines analysing the gut-16S, soil-16S, and soil-ITS datasets. This was estimated by comparing beta diversity between technical replicates (Bray-Curtis distance, BCd and Jaccard distance, Jd). We found that Jd and BCd were the lowest in LotuS2, largely independent of the chosen sequence clustering algorithms and dataset. This indicates a greater reproducibility of community compositions generated by LotuS2 (Fig. 3A, B and Supplementary Figure S2). The lowest BCd and Jd were overall observed for LotuS2-UPARSE (Fig. 3A, B and Supplementary Figure S2) in both gut- and soil-16S datasets, though this was not always significant between different LotuS2 runs (Supplementary Table S2).

Even using the same clustering algorithm, LotuS2-DADA2 compositions were more reproducible compared to both QIIME 2-DADA2 and DADA2 (significant only on soil data). LotuS2-DADA2 denoises by default all reads (per sequencing run) together, while in the default DADA2 setup each sample is denoised separately; the latter strategy has a reduced computational burden but can potentially miss sequence information from rare taxa. Also, LotuS2-VSEARCH compositions were more reproducible than PipeCraft 2-VSEARCH, except in the Jd between the replicates of the soil-16S dataset. mothur showed poorer performance compared to other pipelines on the gut-16S dataset and did not give results for the soil-16S dataset.

We then calculated the fraction of samples being closest in BCd distance to its technical replicate for each pipeline (Fig. 3D, E), simulating the process of identifying technical replicates without prior knowledge. While LotuS1 resulted in the highest fraction of samples being closest to its replicate among all samples in the gut-16S dataset, it performed the worst in the soil-16S dataset. On the other hand, in the mothur result, technical replicates were the most unlikely to be closest to their technical replicate. LotuS2 with UNOISE3 clustering resulted in the highest fraction of samples being closest to its replicate in the soil-16S dataset. When this comparison was made with the non-default options in LotuS2 (using different dereplication parameters, deactivating LULU, using UNCROSS2 or retaining taxonomically unclassified reads), BCd between the technical replicates remained largely unchanged, especially in the soil-16S dataset (Supplementary Figure S2, Supplementary Figure S3A, B and Supplementary information). However, retaining unclassified reads could significantly reduce the reproducibility of LotuS2 results on the gut-16S dataset. Furthermore, even starting the analysis with different read truncation lengths, LotuS2 still had the highest reproducibility in both gut- and soil-16S datasets (Supplementary Figure S4, Supplementary Figure S5 and Supplementary information).

Lastly, we calculated the reproducibility of reported alpha diversity between technical replicate samples in both gut-16S and soil-16S datasets (Supplementary Figure S6A, B). In both datasets, LotuS2 alpha diversity was not significantly different between technical replicates, as expected (6 of 8 comparisons, Wilcoxon signed-rank test). Although this was also the case for PipeCraft 2, in 6 of 6 cases, mothur, QIIME 2, and DADA2 had significant differences in the alpha diversity between technical replicates.

Thus, LotuS2 showed in our benchmarks a higher data usage efficiency and higher reproducibility of community compositions than mothur, PipeCraft 2, QIIME 2, and DADA2. These benchmarks also showed the importance of pre- and post-processing raw reads and OTUs/ASVs, since LotuS2-DADA2 and QIIME 2-DADA2 performed better than DADA2, despite using the same clustering algorithm. LotuS2-VSEARCH also performed better than PipeCraft 2-VSEARCH.

### Benchmarking the soil-ITS dataset

Compared to 16S rRNA gene amplicons, ITS amplicons typically vary more in length [4], thus requiring a different sequence clustering workflow; LotuS2 in ITS mode uses by default CD-HIT to cluster ITS sequences, and ITSx to identify plausible ITS1/2 sequences.

In terms of data usage, both LotuS2 and QIIME 2-DADA2 retrieved similar numbers of reads, but for QIIME 2 these read counts were distributed across twice the number of ASVs (Fig. 2F). QIIME 2-DADA2 reproduced the fungal composition significantly worse in replicate samples, compared to LotuS2-UPARSE, having higher pairwise BCd (Fig. 3C) and Jd (Supplementary Figure S2H, I). However, it spanned the highest fraction of samples closest to its technical replicate, although this fraction was overall very high for all the pipelines (0.978-1) (Fig. 3F). DADA2 showed a poor performance in comparison to the other pipelines, resulting in the lowest data usage efficiency (Fig. 2F) (yielding the highest number of ASVs, lowest retrieved read counts) and the lowest reproducibility (highest BCd) (Fig. 3C, Supplementary Table S2) between replicate samples. LotuS2 had overall the lowest BCd and Jd between replicates, using both UPARSE and CD-HIT clustering (Fig. 3C, Supplementary Figure S2H, I). The use of CD-HIT in combination with ITSx led to increased OTU numbers (from 947 to 1008) although read counts remained mostly the same in the final output matrix and BCd was largely similar (Supplementary Figure S3C). Here, deactivating LULU slightly decreased reproducibility (Supplementary Figure S3C).

Finally, we calculated the reproducibility of alpha diversity between the technical replicate samples in the soil-ITS dataset (Supplementary Figure S6C). All pipelines resulted in no significant difference between the technical replicate samples, thus alpha diversity was reproducible in all pipelines.

### Benchmarking the dataset from the mock microbial community

To assess how well a known community can be reconstructed in LotuS2, we used a previously sequenced 16S mock community [53] containing 43 genera and 59 microbial strains, where complete reference genomes were available.

**Fig. 4:**
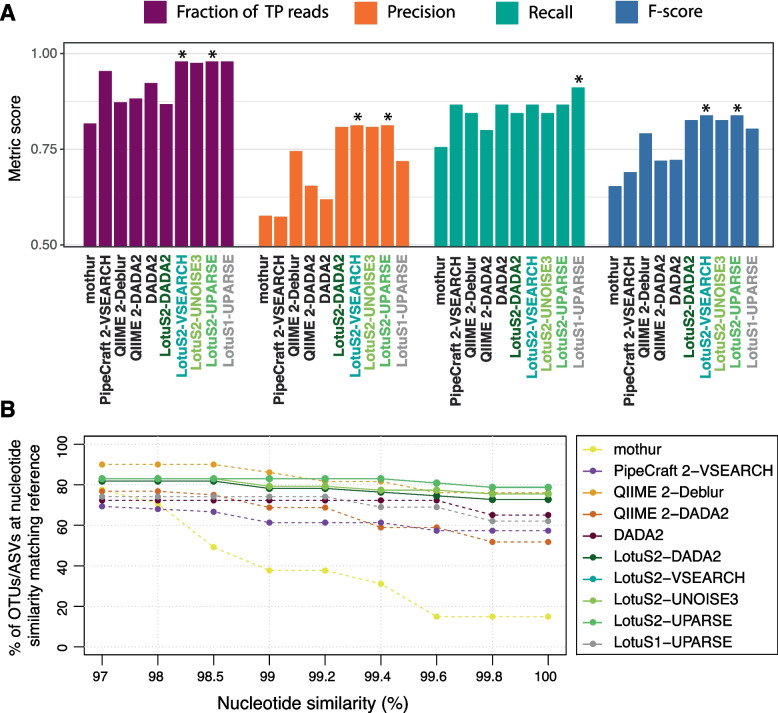
Benchmarking of amplicon sequence data analysis pipeline’s performance using a mock community with known species composition. **A** Accuracy of each pipeline in predicting the mock community composition at genus level. For benchmarking we compared the fraction of reads assigned to true genera and both correctly and erroneously recovered genera. Precision, Recall, and F-score were calculated based on the true positive, false positive, and false negative taxa identified. At species level, LotuS2 excelled also in these statistics (Supplementary Figure S9). **B** Percentage of true positive ASVs/OTUs having a nucleotide identity ≥ indicated thresholds to 16S rRNA gene sequences of genomes from the mock community. Pipeline(s) showing the highest performance in each comparison is denoted with a star (*). TP, true positive; ASV, amplicon sequencing variant; OTU, operational taxonomic unit. LotuS2-UPARSE and LotuS2-VSEARCH had the same result, therefore colors are overlaid

All pipelines performed poorly at reconstructing the community composition (Pearson *R* = 0.43–0.67, Spearman Rho = 0.54–0.80, Supplementary Table S3 and Supplementary Figure S7), possibly related to PCR biases and rRNA gene copy number variation. Therefore, we focused on the number of correctly identified taxa. For this, we calculated the number of reads assigned to true taxa as well as precision, recall, and *F*-score at genus level. LotuS2-VSEARCH and LotuS2-UPARSE had the highest precision, *F*-score, and fraction of reads assigned true positive taxa, (Fig. 4A and Supplementary Figure S8). LotuS1 had the highest recall, but low precision. When applying the same tests at species level, LotuS2-DADA2 had overall the highest precision and F-score (Supplementary Figure S9). QIIME 2-Deblur had often competitive, but slightly lower, precision, recall, and *F*-scores compared to LotuS2, while mothur, PipeCraft 2-VSEARCH, QIIME 2-DADA2, and DADA2 scores were lower (Fig. 4A).

Next, we investigated which software could best report the correct OTU/ASV sequences. For this, we calculated the fraction of TP OTUs/ASVs (i.e., OTUs/ASVs which are assigned to a species based on the custom mock reference taxonomy) with 97–100% nucleotide identity to 16S rRNA sequences from reference genomes in each pipeline (Fig. 4B). Here, LotuS2-VSEARCH and LotuS2-UPARSE reported OTU sequences were most often identical to the expected sequences, having 82.2% of the OTU sequences at 100% nucleotide identity to reference sequences. QIIME 2-Deblur ASV sequences were of similar quality, but slightly less often at 100% nucleotide identity (78.2%). DADA2, QIIME 2-DADA2 and PipeCraft 2-VSEARCH ASV/OTU sequences were often more dissimilar to the expected reference sequences. It is noteworthy that LotuS2-DADA2 and LotuS2-VSEARCH outperformed these pipelines based on the same sequence clustering algorithm, likely related to the stringent read filtering and seed extension step in LotuS2.

The mock community consisted of 49 bacteria and 10 archaea [53], with a total of 128 16S rRNA gene copies included in their genomes. If multiple 16S copies occur within a single genome, these can diverge but are mostly highly similar or even identical to each other [57]. Thus, the expected biodiversity would be 59 OTUs and ≤ 128 ASVs. Notably, the number of mothur and QIIME 2-Deblur TP ASVs/OTUs exceeded this threshold (*N* = 370, 198, respectively), indicating that both pipelines overestimate known biodiversity. DADA2, QIIME 2-DADA2, and PipeCraft 2-VSEARCH generated more ASVs than expected per species (*N* = 94, 122, and 90 respectively), but this might be explained by divergent within-genome 16S rRNA gene copies. LotuS2 was notably at the lower end in predicted biodiversity, predicting between 53 and 61 OTUs or ASVs in different clustering algorithms (Supplementary Table S4). However, these seemed to mostly represent single species, covering the present species best among pipelines, as the precision at species level was highest for LotuS2 (Supplementary Figure S9), thus capturing species level biodiversity most accurately.

Based on the mock community data, LotuS2 was more precise in the reported 16S rRNA gene sequences, assigning the correct taxonomy, and detecting biodiversity. Within-genome 16S copies were less likely to be clustered separately using LotuS2.

## Discussion

LotuS2 offers a fast, accurate, and streamlined amplicon data analysis with new features and substantial improvements since LotuS1. Software and workflow optimizations make LotuS2 substantially faster than all QIIME 2, DADA2, and mothur. On large datasets, this advantage becomes crucial for users: for example, we processed a highly diverse soil dataset consisting of > 11 million non-demultiplexed PacBio HiFi amplicons (26 Sequel II libraries) in 2.5 days on 16 CPU cores, using a single command (unpublished data). Besides being more resource and user-friendly, compositional matrices from LotuS2 were more reproducible and accurate across all tested datasets (gut 16S, soil 16S, soil ITS, and mock community 16S).

LotuS2 owes high reproducibility and accuracy to the efficient use of reads based on their quality tiers in different steps of the pipeline. Low-quality reads introduce noise and can artificially inflate observed biodiversity, i.e., the number of OTUs/ASVs [58]. Conversely, an overly strict read filter will decrease sensitivity for low-abundant members of a community by artificially reducing sequencing depth. To find a trade-off, LotuS2 uses only truncated, high-quality reads for sequence clustering (except ITS amplicons), while the read backmapping and seed extension steps restore some of the discarded sequence data.

Notably, OTUs/ASVs reported with LotuS2 were the most similar (at > 99% identity) to the reference, compared to other pipelines (Fig. 4B). This was mostly independent of clustering algorithms used, rather resulting from a combination of selecting high-quality reads for sequence clustering and the seed extension step selecting a high-quality read (pair) best representing each OTU or ASV. The seed extension unique to LotuS2 also decouples read clustering and read merging, avoiding the use of the error-prone 3′ read end or the second read pair during the error sensitive sequence clustering step [18]. Decoupling sequence clustering length restrictions from other pipeline steps thus avoids limiting information in computational steps benefitting from longer DNA sequences, such as taxonomic assignments or phylogeny reconstructions.

In conclusion, LotuS2 is a major improvement over LotuS1, representing pipeline updates that accumulated over the past 8 years. It offers superior computational performance, accuracy, and reproducibility of results, compared to the other tested pipelines. Importantly, it is straightforward to install, and programmed to reduce required user time and knowledge, following the idea that “less is more with LotuS2”.

## Supplementary Information

**Additional file 1: Supplementary Table S1.** Read counts and number of OTUs/ASVs in the OTU/ASV matrix of each pipeline.

**Additional file 2: Supplementary Table S2.** Significance of differences between each pipeline in the reproducibility of beta diversity between the technical replicates. Significance of differences in Bray-Curtis distance between the pipelines were calculated based on the Tukey’s HSD test.

**Additional file 3: Supplementary Table S3.** Correlation and beta distance between the mock community and re-constructed mock community by each pipeline. A-B) Spearman and Pearson correlation between the expected abundances in the mock community and the observed abundances by each pipeline. C) Bray-Curtis dissimilarity between the known mock community and re-constructed mock community composition by each pipeline.

**Additional file 4: Supplementary Table S4.** Accuracy of each pipeline in re-constructing the mock community at genus level.

**Additional file 5: Supplementary Figure S1.** Galaxy web interface of LotuS2. Raw reads can be uploaded into the LotuS2 via the Galaxy web interface and analysed (accessible on https://usegalaxy.eu/).

**Additional file 6: Supplementary Figure S2.** Reproducibility and data usage efficiency respective to dereplication filtering. A, D and G) Data usage efficiency of each tested pipeline at different dereplication parameters of LotuS2 (from strictest to least strict dereplication: 20:1,12:3,6:2; 15:1,9:3,12:2; 10:1,6:3,8:2; 8:1,4:2,3:3 (default); 4:1; 2:1, and 1:1) using DADA2 or CD-HIT clustering for 16S and ITS datasets, respectively, by comparing the number of sequence clusters (OTUs/ASVs) to retrieved read counts in final output matrix.The dereplication can be fine controlled through a syntax. For example, 8:1,4:2,3:3 means that a read is accepted, if it occurs >=8 times in >= 1 samples or >4 times total in >= 2 samples or >=3 times in >= 3 samples.

**Additional file 7: Supplementary Figure S3.** Reproducibility of the technical replicates respective to different LotuS2 non-default parameters. Bray-Curtis distances between technical replicates of A) gut-16S, B) soil-16S, and C) soil-ITS datasets using default and non-default parameters (LotuS2 flags: -lulu 0, -xtalk 1, -keepUnclassified 1, -ITSx 0, where 1 means the option is activated; 0 means deactivated). When activated, -lulu option uses LULU R package [24] to merge OTUs/ASVs based on their co-occurrences; -xtalk option checks for cross-talk [33], -keepUnclassified includes unclassified (i.e. not matching to any taxon in the taxonomy database) OTUs/ASVs in the final matrix and –ITSx activates the ITSx program [32] to only retain OTUs fitting to ITS1/ITS2 hmm models.

**Additional file 8: Supplementary Figure S4.** Data usage efficiency of different amplicon sequence data analysis pipelines. Data usage efficiency on gut 16S rRNA (gut- 16S) and soil 16S rRNA (soil-16S) amplicons tested with different pipelines at different read truncation lengths (170, 200, and 230 & 170, 200, and 220 bases for the gut and soil datasets, respectively), by comparing the number of sequence clusters (ASVs /OTUs) to retrieved read counts in the final output matrix of each pipeline. In all other analysis, default values were used for LotuS2 (200 bases).

**Additional file 9: Supplementary Figure S5.** Reproducibility of beta diversity at different read truncation lengths. Reproducibility of sequenced technical replicates by measuring the Bray-Curtis (A and C) and Jaccard distances (B and D) of the microbiome composition among technical replicate samples. Two datasets were used to represent different biomes and amplicon technologies using (A, B) human faecal samples (16S rRNA primer, N=40 replicates) and (C, D) soil samples (16S rRNA, V4-V5 region primers, N=50 replicates). Lower Bray-Curtis or Jaccard distances between technical replicates indicate better reproducibility of community compositions. Default pipeline parameters and recommended settings for each dataset were used (Please see the Supplementary information for further information).

**Additional file 10: Supplementary Figure S6.** Reproducibility of alpha diversity between technical replicates. OTU/ASV richness was calculated for A) gut-16S, B) soil-16S, and C) soil-ITS datasets. Samples were rarefied to an equal number of reads per sample before calculating richness, and any samples whose replicate pair was removed after rarefaction (because of having lower number of reads than the rarefaction depth) were excluded from further analysis. LotuS1 results for soil-16S were removed due to too many samples being removed in rarefactions. Significance of differences in richness between the sets were calculated based on the paired samples Wilcoxon test (***, **, *, and “ns” denotes p<0.0005, p<0.005, p<0.05, and p> 0.05 (i.e., not significant), respectively).

**Additional file 11: Supplementary Figure S7.** Observed composition of the mock community compared to the composition predicted by each pipeline. A) Relative abundances of the 16 orders having the highest abundance. B) Bray-Curtis distance based PCoA of the observed composition of the mock sample and composition predicted by each pipeline

**Additional file 12: Supplementary Figure S8.** Number of reads and OTUs/ASVs and those assigned true taxa at genus level by each pipeline in the analysis of the mock community. Total number of A) reads retrieved by each pipeline and those assigned to true taxa at genus level B) OTUs/ASVs generated by each pipeline and those assigned to true taxa at genus level. Blue and red line indicates number of 16S gene copies and species, respectively, in the mock community.

**Additional file 13: Supplementary Figure S9.** Accuracy of each pipeline in predicting the mock community composition at species level. For benchmarking we compared the fraction of reads assigned to true taxa and both correctly and erroneously recovered taxa at the species level from the mock community.

**Additional file 14: Supplementary information.**

## Data Availability

The datasets generated during the current study are available here: https://zenodo.org/record/6857189#.YtWSRXbMI2w Mock-16 community is downloaded from the *mockrobiota* repository [53]: https://s3-us-west-2.amazonaws.com/mockrobiota/latest/mock-16/mock-forward-read.fastq.gz https://s3-us-west-2.amazonaws.com/mockrobiota/latest/mock-16/mock-reverse-read.fastq.gz Availability of LotuS2: Documentation, tutorials: lotus2.earlham.ac.uk, Installation via bioconda: https://anaconda.org/bioconda/lotus2 Galaxy wrapper (MIT licensed): https://github.com/TGAC/earlham-galaxytools/tree/master/tools/lotus2 and https://toolshed.g2.bx.psu.edu/view/earlhaminst/lotus2/ Galaxy server: https://usegalaxy.eu/ Programs (GPLv3 licensed): https://github.com/hildebra/lotus2, https://github.com/hildebra/sdm, https://github.com/hildebra/LCA LotuS2 users can report an issue or propose a new feature by opening a new issue in the LotuS2 github repository. All the commands used for the benchmarking are available in https://github.com/ozkurt/lotus2_benchmarking

## Abbreviations

OTU: Operational taxonomic unit
ASV: Amplicon sequence variant
ITS: Internal transcribed spacer
TP: True positive
FN: False negative
FP: False positive
LotuS: Less OTU Scripts
sdm: Simple demultiplexer
LCA: Least common ancestor
DADA: The Divisive Amplicon Denoising Algorithm
QIIME: Quantitative Insights Into Microbial Ecology

## Footnotes

1 Note that UNOISE3 uses the term zero-range OTUs (zOTUs); for brevity, this is omitted throughout the text.

## Declarations

Ethics approval and consent to participate
