# Supplementary material for: LotuS2: an ultrafast and highly accurate tool for amplicon sequencing analysis
Source: Microbiome. 2022 Oct 19;10:176. doi: 10.1186/s40168-022-01365-1 (PMC9580208; doi:10.1186/s40168-022-01365-1)
Supplement: Supplementary file 6 — Additional file 5: Supplementary Figure S1. Galaxy web interface of LotuS2. Raw reads can be uploaded into the LotuS2 via the Galaxy web interface and analysed (accessible on https://usegalaxy.eu/). [file 40168_2022_1365_MOESM5_ESM.zip › 1-Supp Figure 1.pdf]

TP service! Easier and faster. Instruction on **https://galaxyproject.eu/ftp**

LotusS2 fast OTU processing pipeline (Galaxy Version 2.09.2)

Single- or Paired-end data?

Single-end

! Please provide a value for this option.

Single-end reads

No fastqsanger or fastqsanger.gz dataset available.

Mapping file (optional)

No tabular dataset available.

Needed to demultiplex the FASTQ files using sdm. If the FASTQ are already demultiplexed, this can be omitted. (-map)

SDM option file (optional)

No txt dataset available.

(-sdmopt)

Sequencing platform

(Default)

(-platform)

Barcode (MID) sequences (optional)

No fastqsanger dataset available.

FASTQ file with barcodes (in the processed mi/hiSeq format), if provided by the sequencer (-barcode)

Forward primer used to amplify DNA region (optional)

E.g. 16S primer fwd (-forwardPrimer)

Reverse primer used to amplify DNA region (optional)

E.g. 16S primer rev (-reversePrimer)

Remove likely contaminant OTUs/ASVs based on alignment to host genome

Disabled

Useful for low-bacterial biomass samples to remove possible host genome contaminations (-offtargetDB)

Clustering Options

Taxonomy Options

Execute
