# Supplementary figures and images for: LotuS2: an ultrafast and highly accurate tool for amplicon sequencing analysis
Source: Microbiome. 2022 Oct 19;10:176. doi: 10.1186/s40168-022-01365-1 (PMC9580208; doi:10.1186/s40168-022-01365-1)

## GUT 16S rRNA

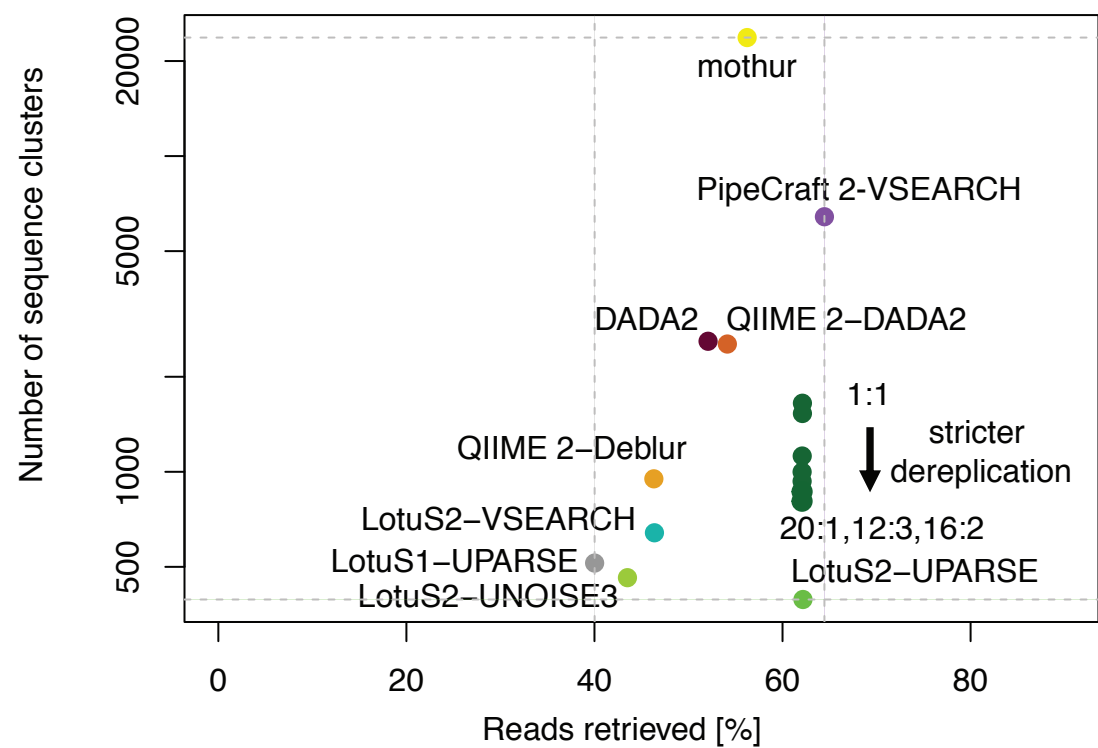

**B**

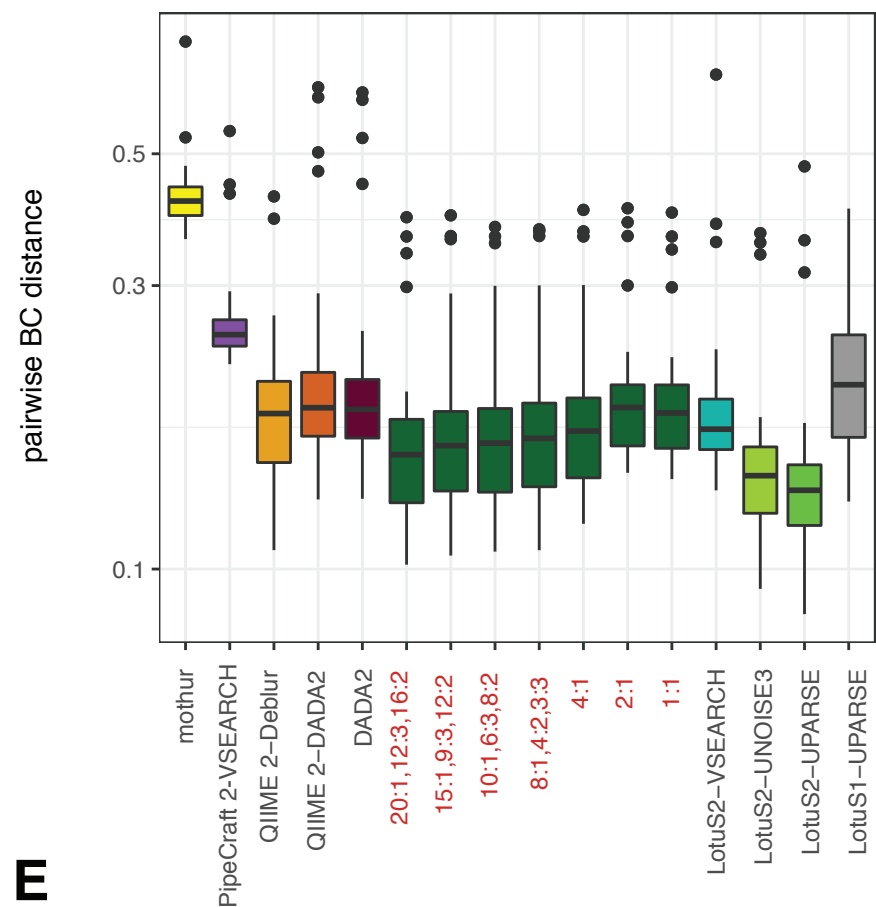

**C**

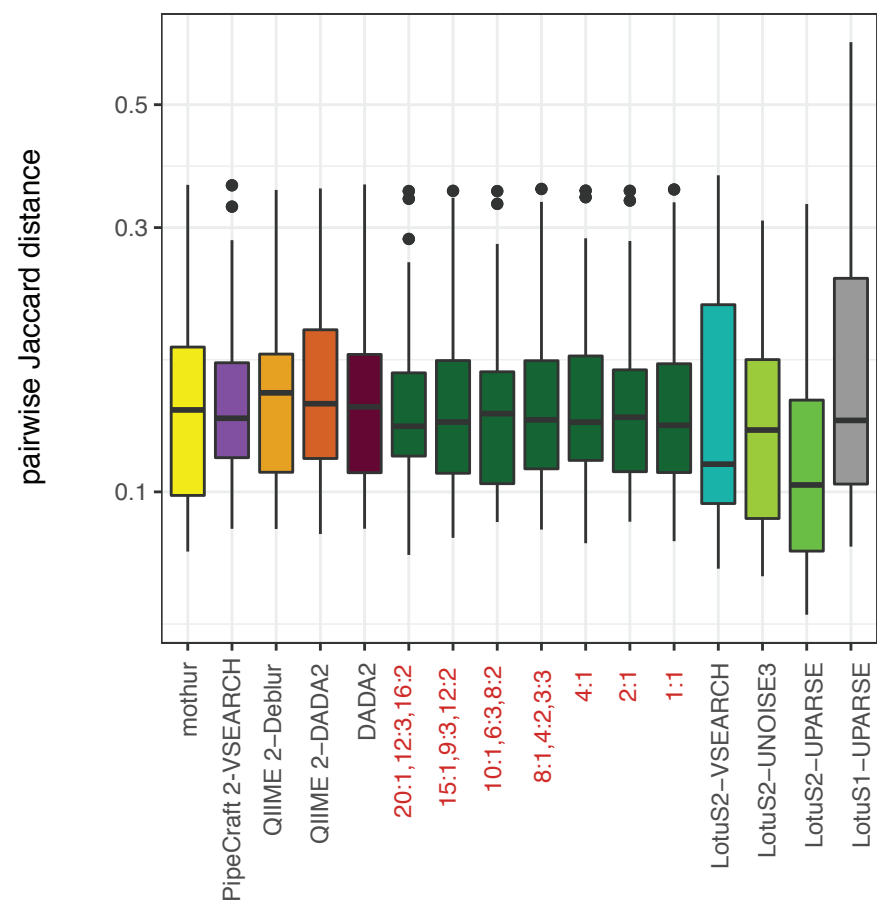

## SOIL 16S rRNA

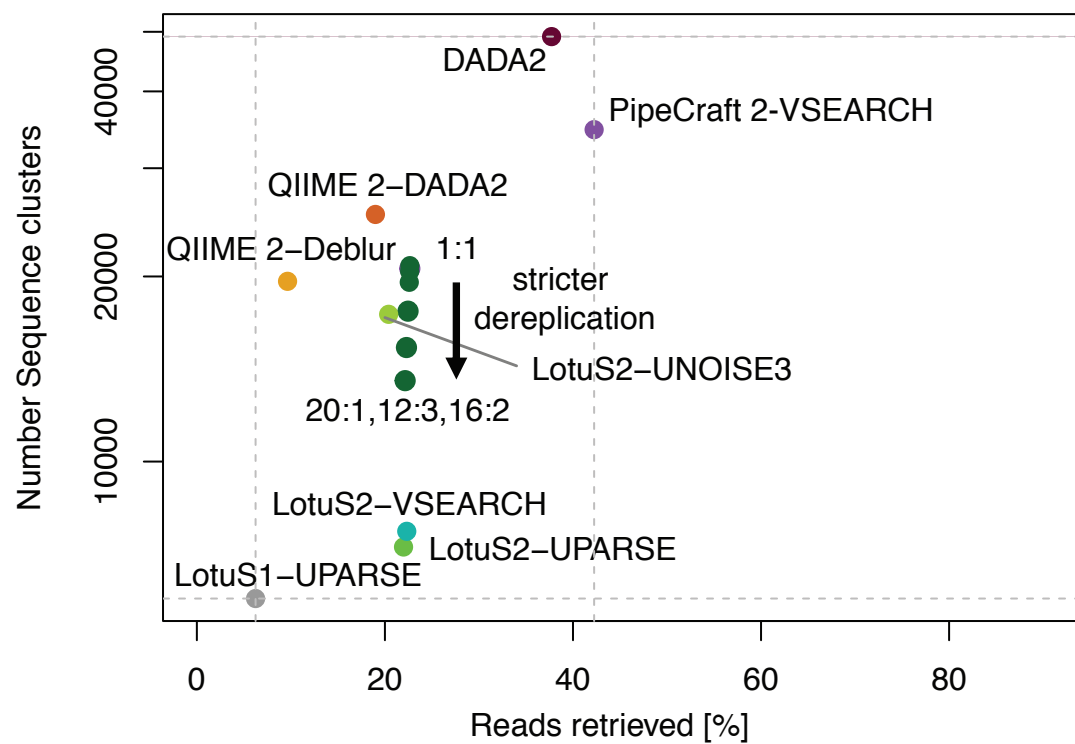

**E**

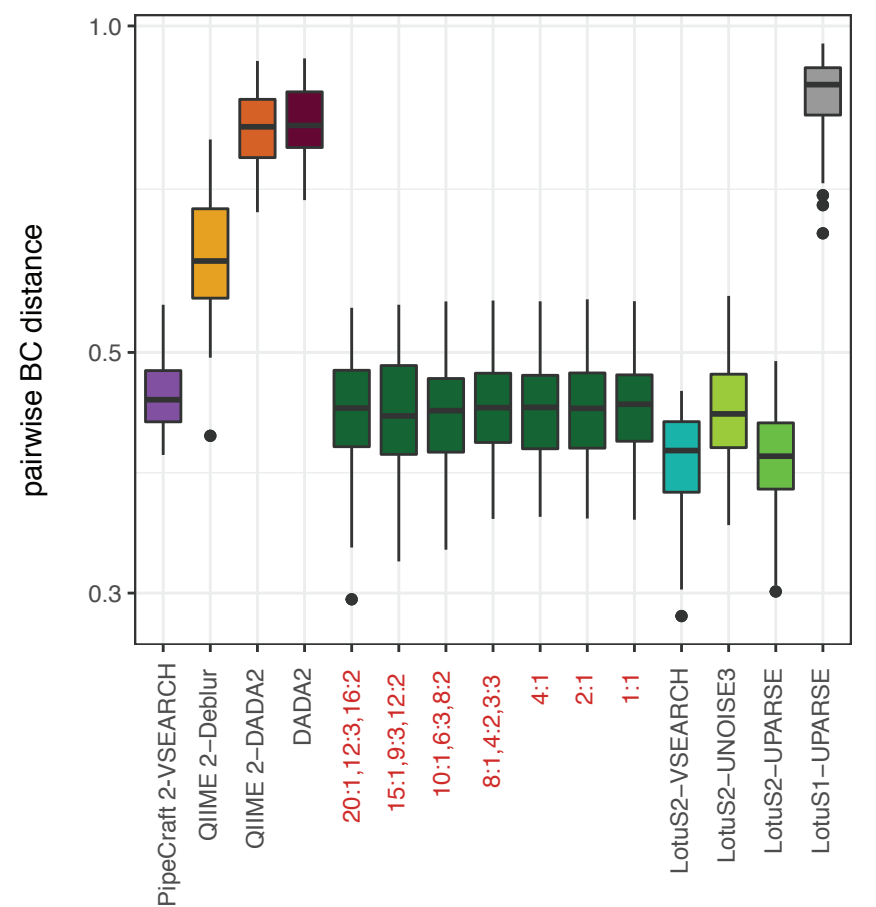

**F**

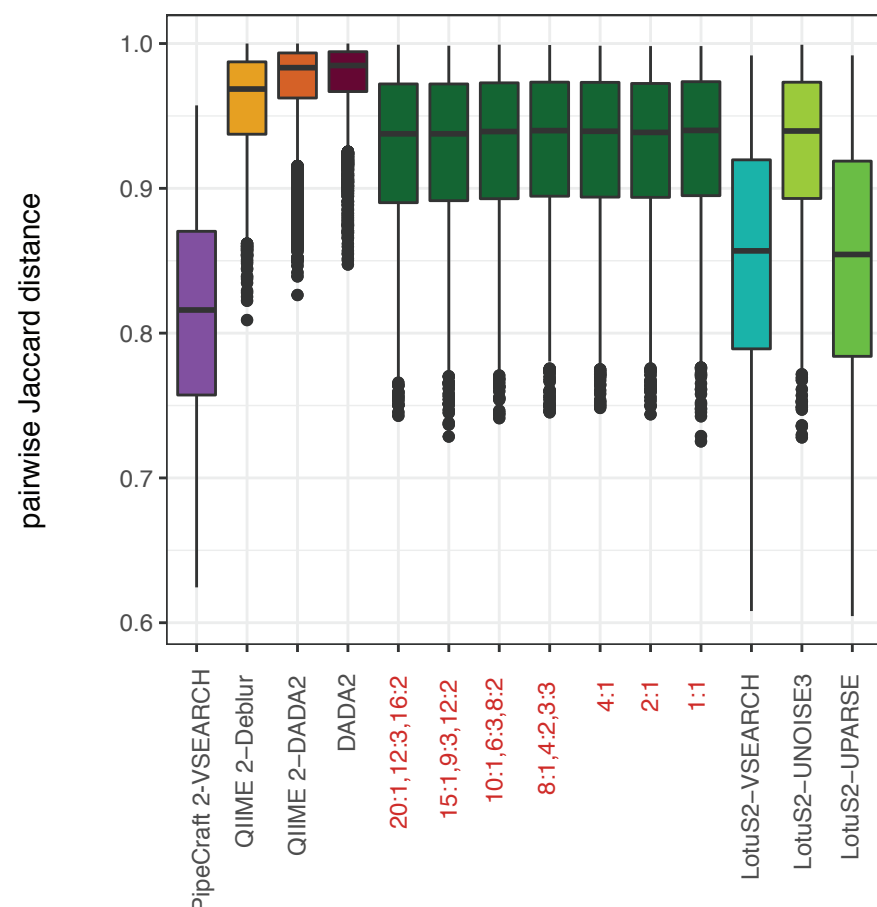

**G**

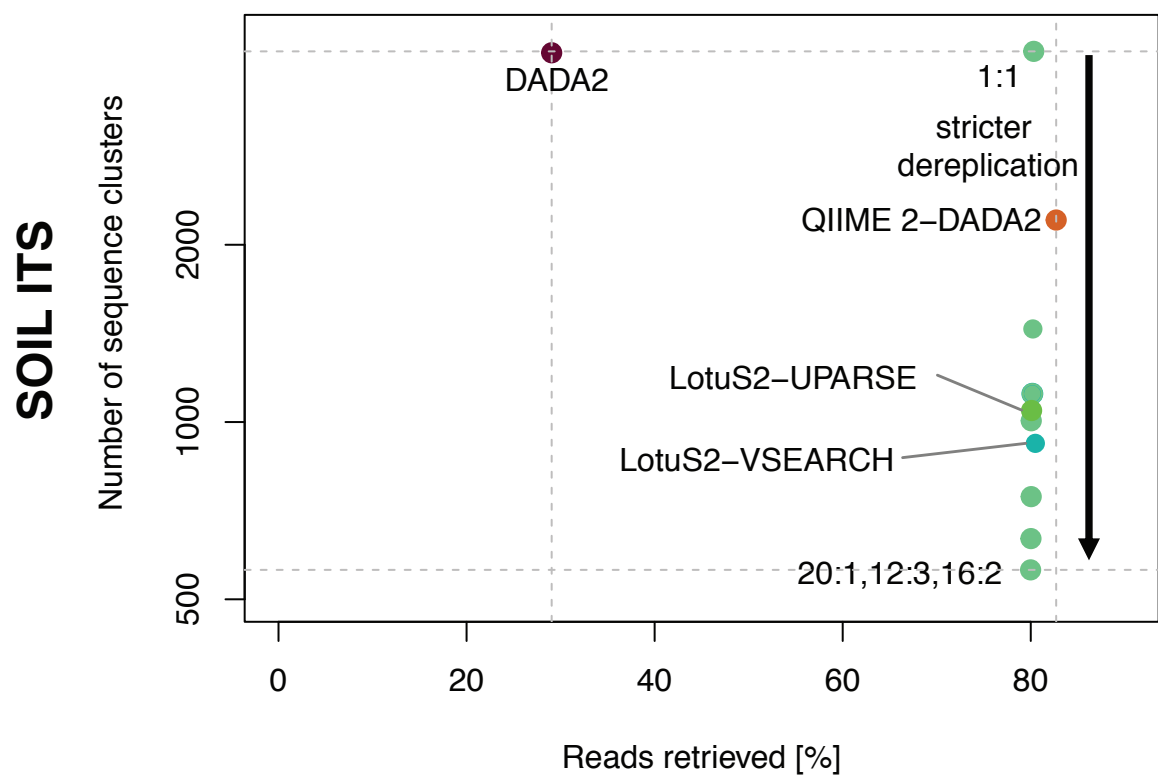

**H**

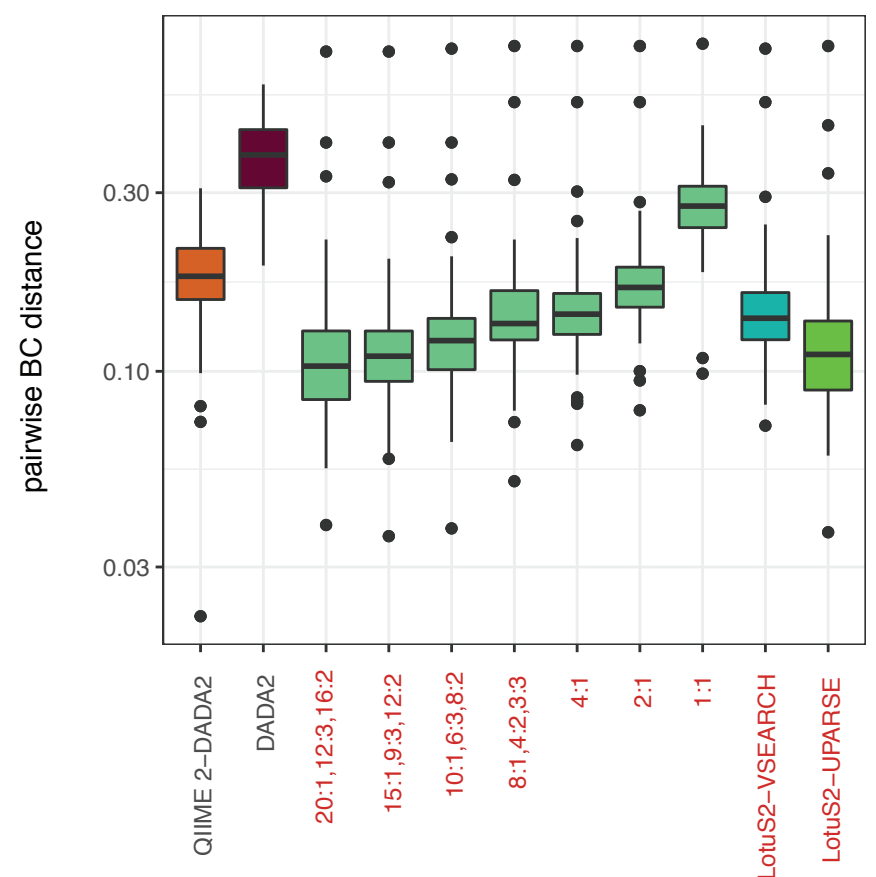

**I**

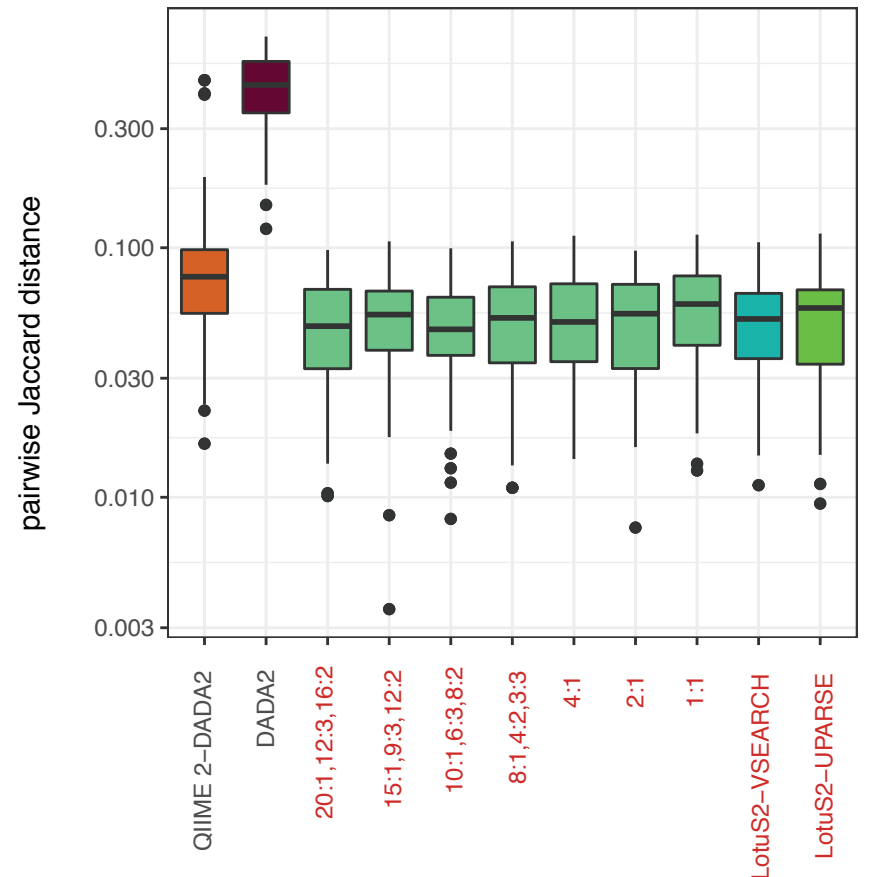

Supplement: Supplementary file 7 — Additional file 6: Supplementary Figure S2. Reproducibility and data usage efficiency respective to dereplication filtering. A, D and G) Data usage efficiency of each tested pipeline at different dereplication parameters of LotuS2 (from strictest to least strict dereplication: 20:1,12:3,6:2; 15:1,9:3,12:2; 10:1,6:3,8:2; 8:1,4:2,3:3 (default); 4:1; 2:1, and 1:1) using DADA2 or CD-HIT clustering for 16S and ITS datasets, respectively, by comparing the number of sequence clusters (OTUs/ASVs) to retrieved read counts in final output matrix.The dereplication can be fine controlled through a syntax. For example, 8:1,4:2,3:3 means that a read is accepted, if it occurs >=8 times in >= 1 samples or >4 times total in >= 2 samples or >=3 times in >= 3 samples. [file 40168_2022_1365_MOESM6_ESM.pdf]

**A****GUT 16S rRNA**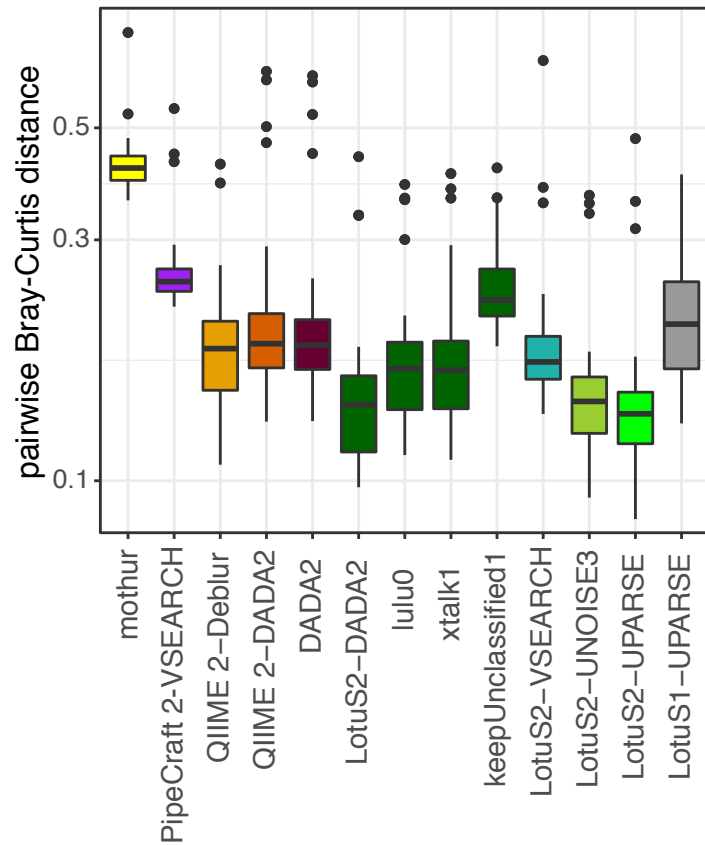**B****SOIL 16S rRNA**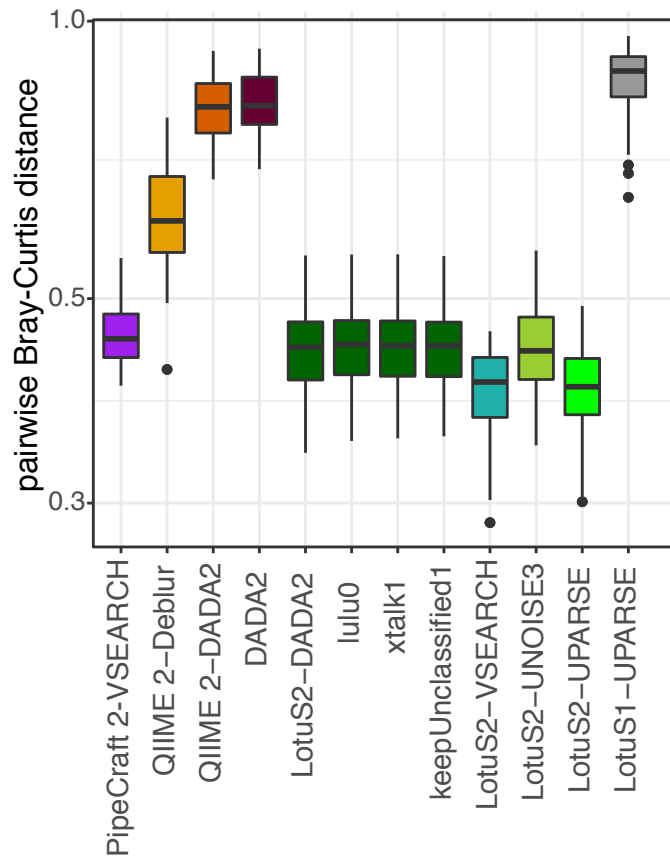**C****SOIL ITS**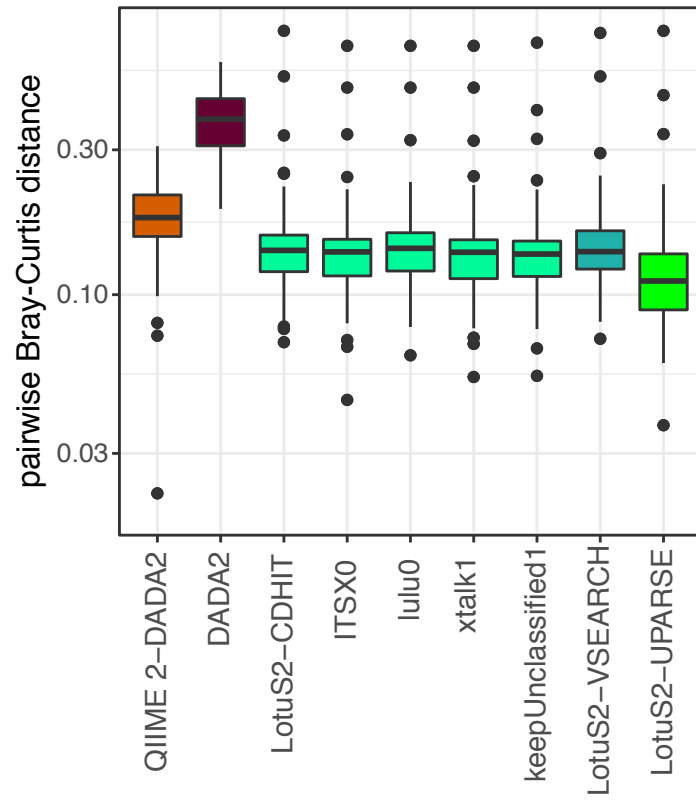

Supplement: Supplementary file 8 — Additional file 7: Supplementary Figure S3. Reproducibility of the technical replicates respective to different LotuS2 non-default parameters. Bray-Curtis distances between technical replicates of A) gut-16S, B) soil-16S, and C) soil-ITS datasets using default and non-default parameters (LotuS2 flags: -lulu 0, -xtalk 1, -keepUnclassified 1, -ITSx 0, where 1 means the option is activated; 0 means deactivated). When activated, -lulu option uses LULU R package [24] to merge OTUs/ASVs based on their co-occurrences; -xtalk option checks for cross-talk [33], -keepUnclassified includes unclassified (i.e. not matching to any taxon in the taxonomy database) OTUs/ASVs in the final matrix and –ITSx activates the ITSx program [32] to only retain OTUs fitting to ITS1/ITS2 hmm models. [file 40168_2022_1365_MOESM7_ESM.pdf]

**A****170 bases**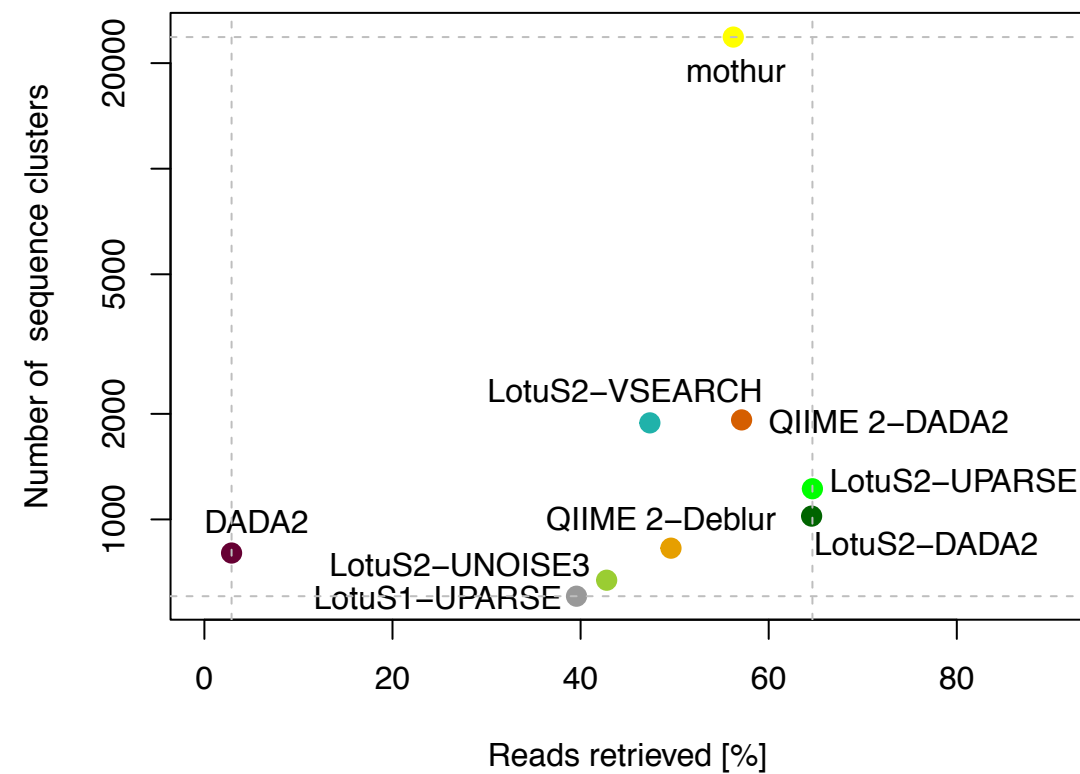**B****200 bases**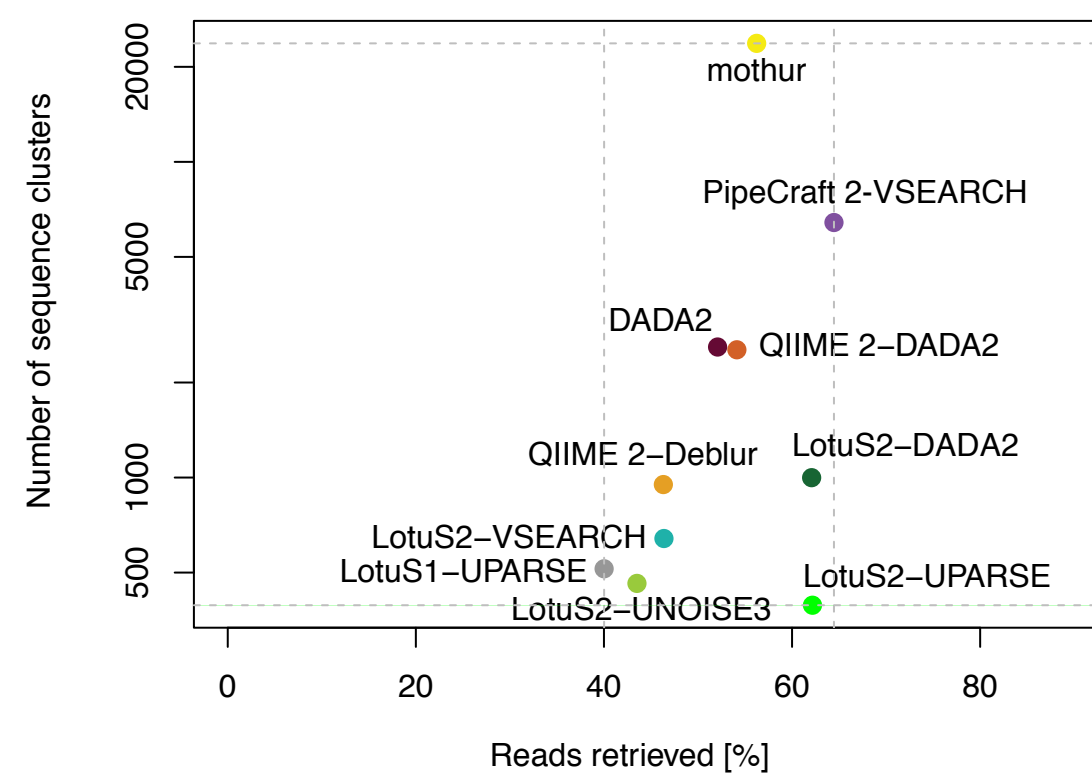**C****230 bases**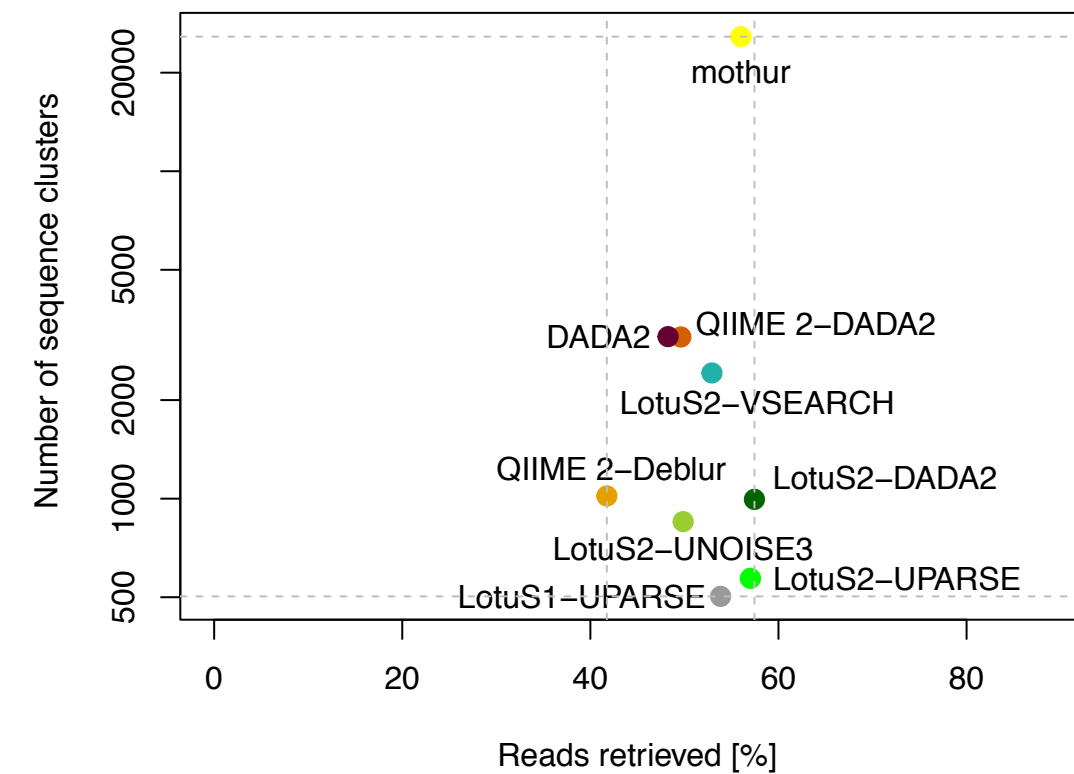**D****170 bases**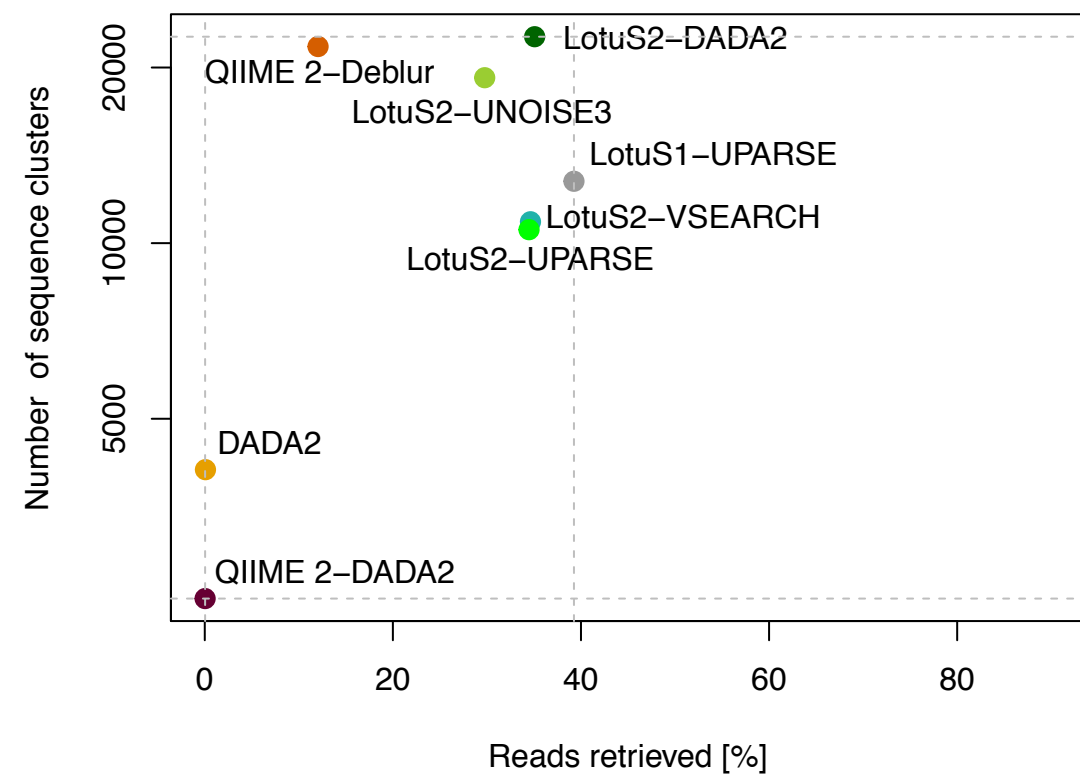**E****200 bases**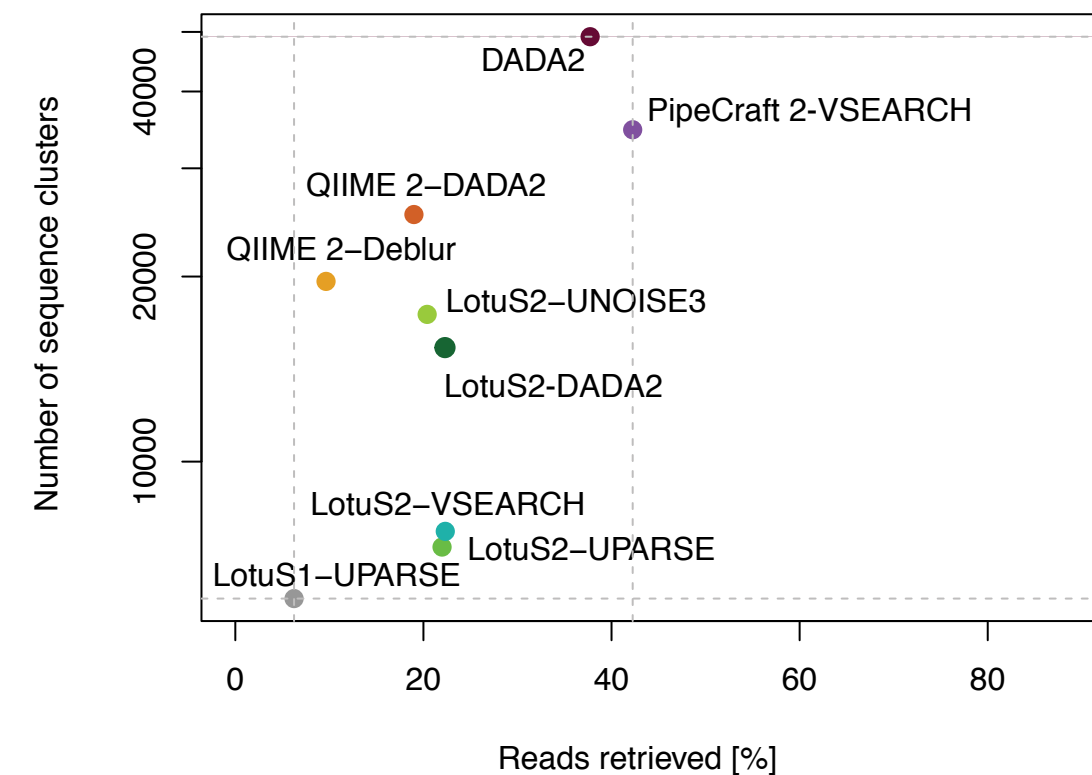**F****220 bases**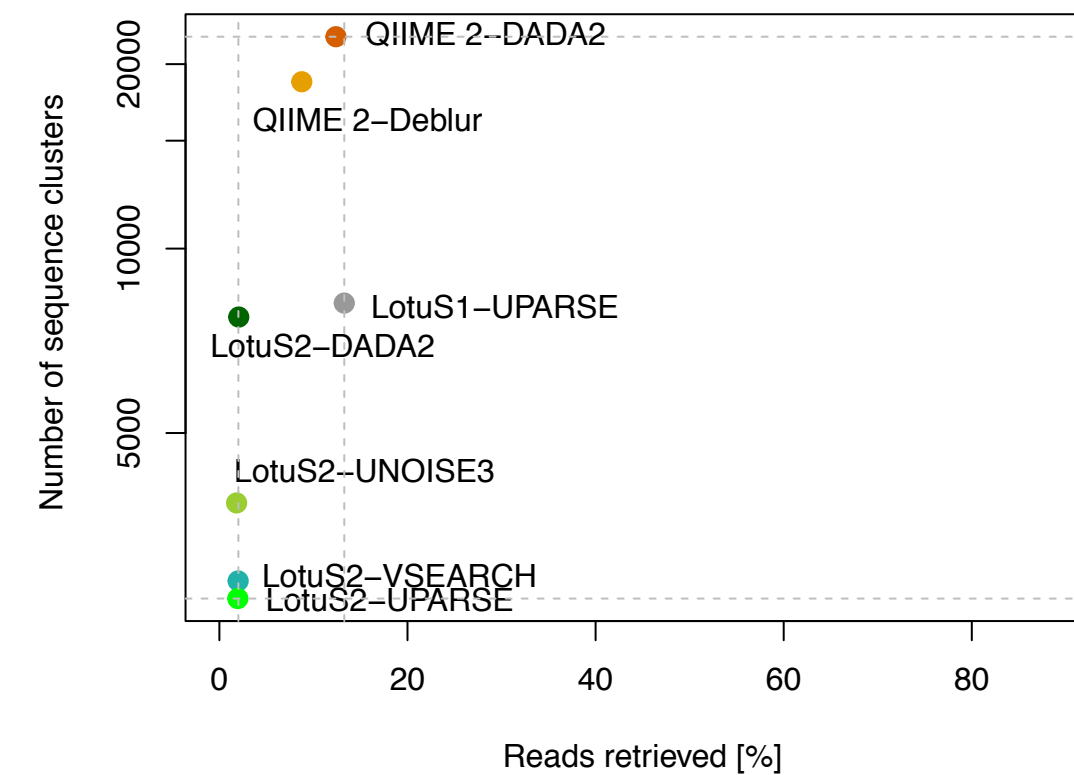

Supplement: Supplementary file 9 — Additional file 8: Supplementary Figure S4. Data usage efficiency of different amplicon sequence data analysis pipelines. Data usage efficiency on gut 16S rRNA (gut- 16S) and soil 16S rRNA (soil-16S) amplicons tested with different pipelines at different read truncation lengths (170, 200, and 230 & 170, 200, and 220 bases for the gut and soil datasets, respectively), by comparing the number of sequence clusters (ASVs /OTUs) to retrieved read counts in the final output matrix of each pipeline. In all other analysis, default values were used for LotuS2 (200 bases). [file 40168_2022_1365_MOESM8_ESM.pdf]

A

## GUT 16S rRNA

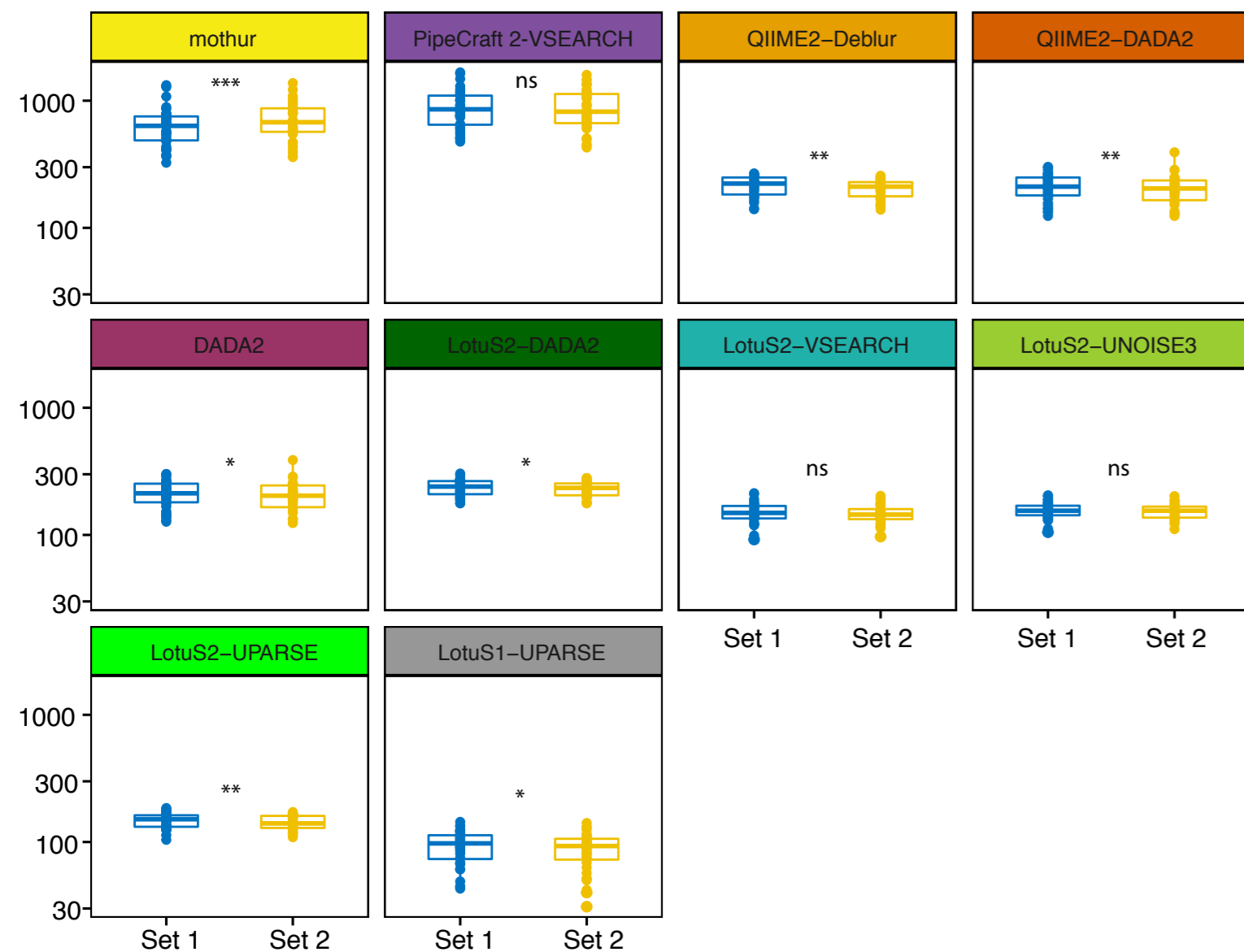

B

## SOIL 16S rRNA

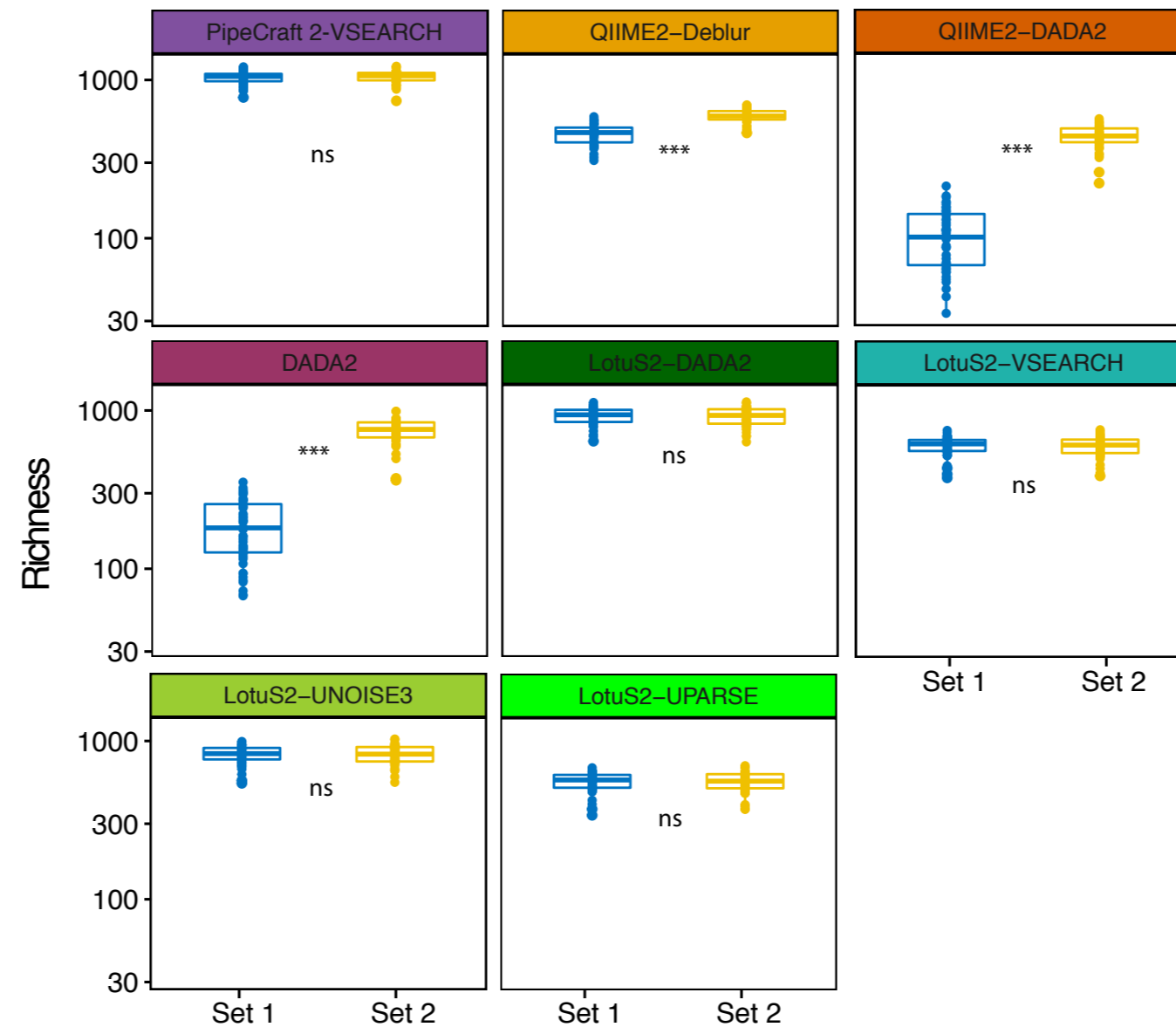

C

## SOIL ITS

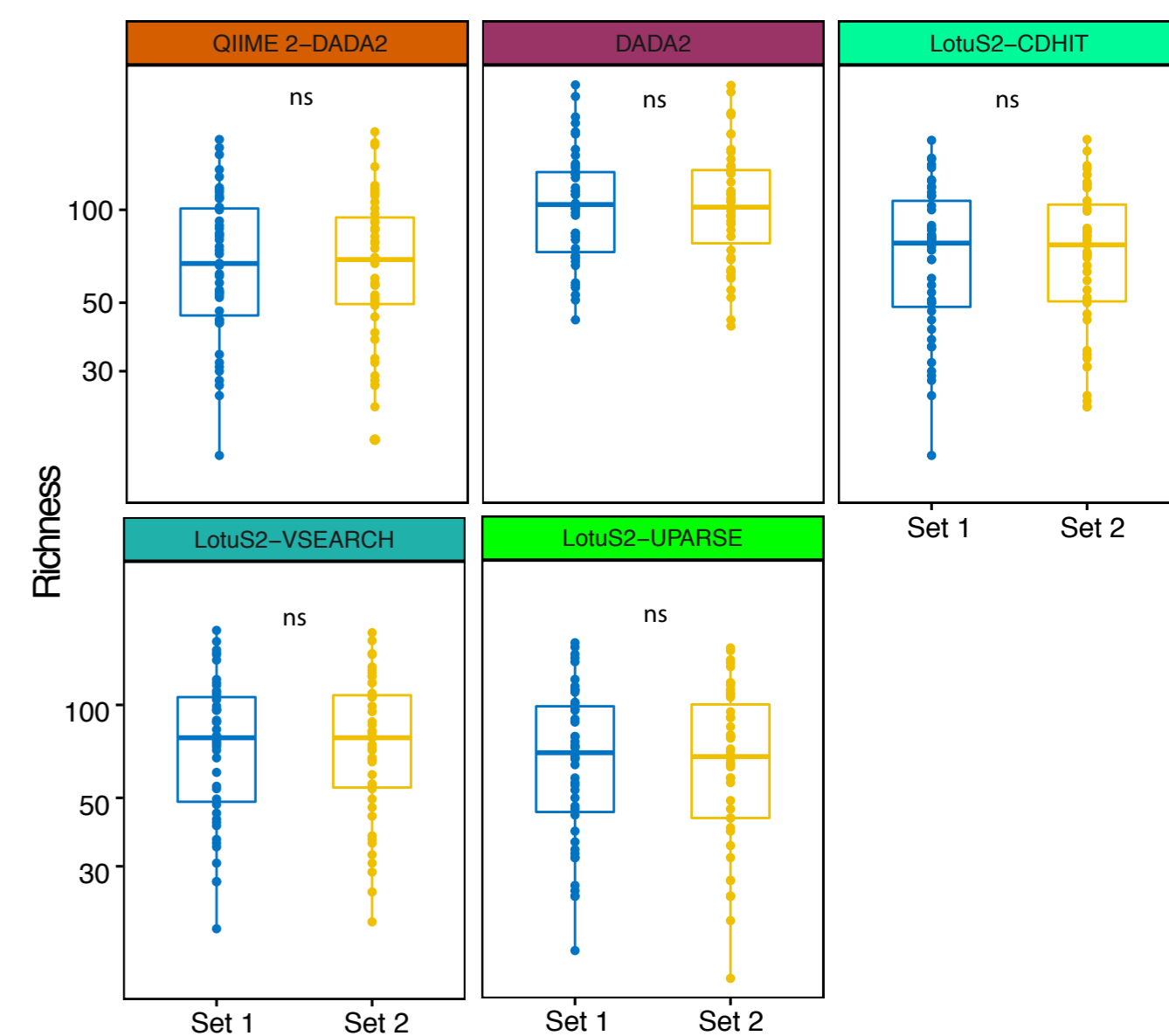

Technical Replicates Set 1 Set 2

Supplement: Supplementary file 11 — Additional file 10: Supplementary Figure S6. Reproducibility of alpha diversity between technical replicates. OTU/ASV richness was calculated for A) gut-16S, B) soil-16S, and C) soil-ITS datasets. Samples were rarefied to an equal number of reads per sample before calculating richness, and any samples whose replicate pair was removed after rarefaction (because of having lower number of reads than the rarefaction depth) were excluded from further analysis. LotuS1 results for soil-16S were removed due to too many samples being removed in rarefactions. Significance of differences in richness between the sets were calculated based on the paired samples Wilcoxon test (***, **, *, and “ns” denotes p<0.0005, p<0.005, p<0.05, and p> 0.05 (i.e., not significant), respectively). [file 40168_2022_1365_MOESM10_ESM.pdf]

**A**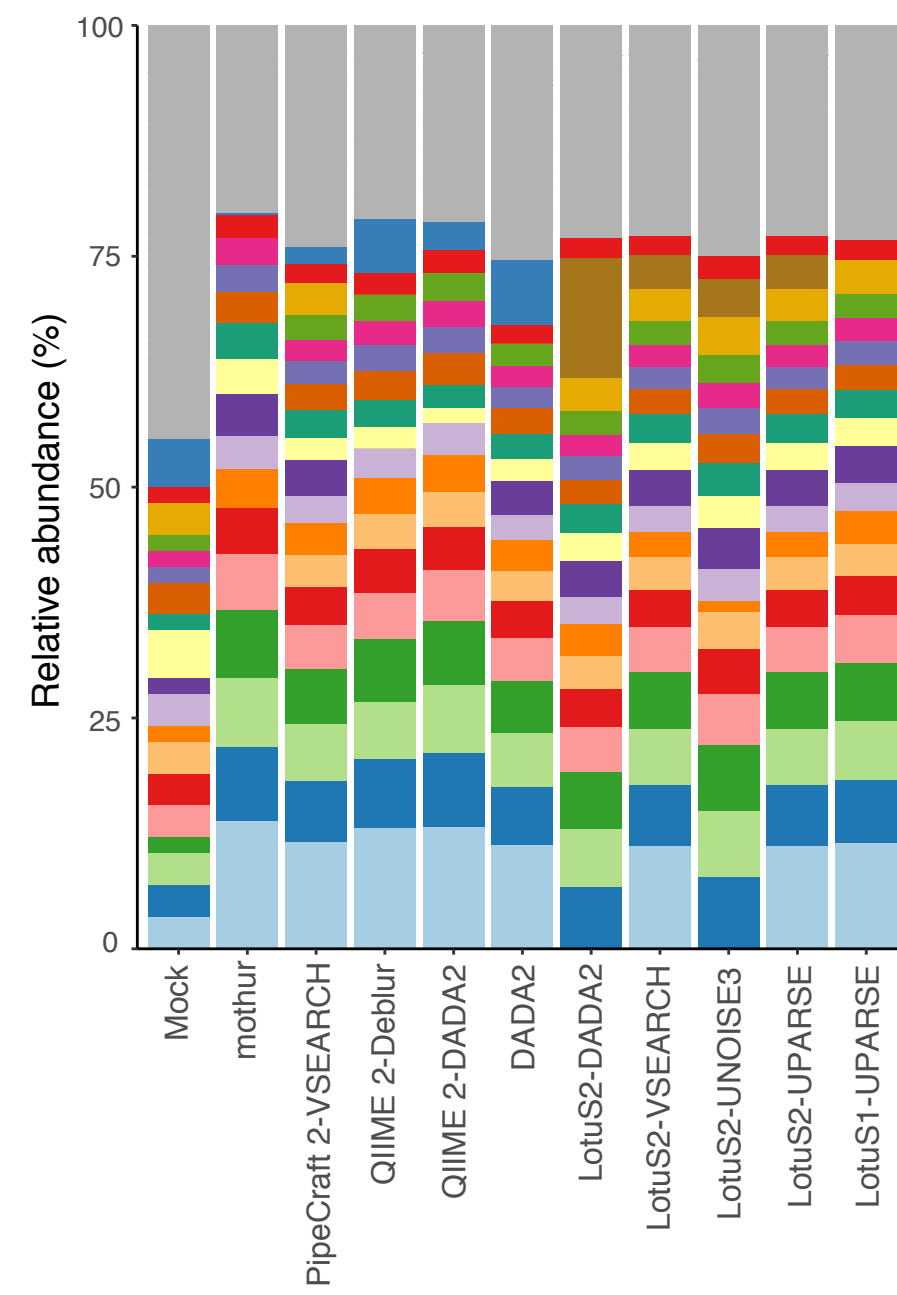**B**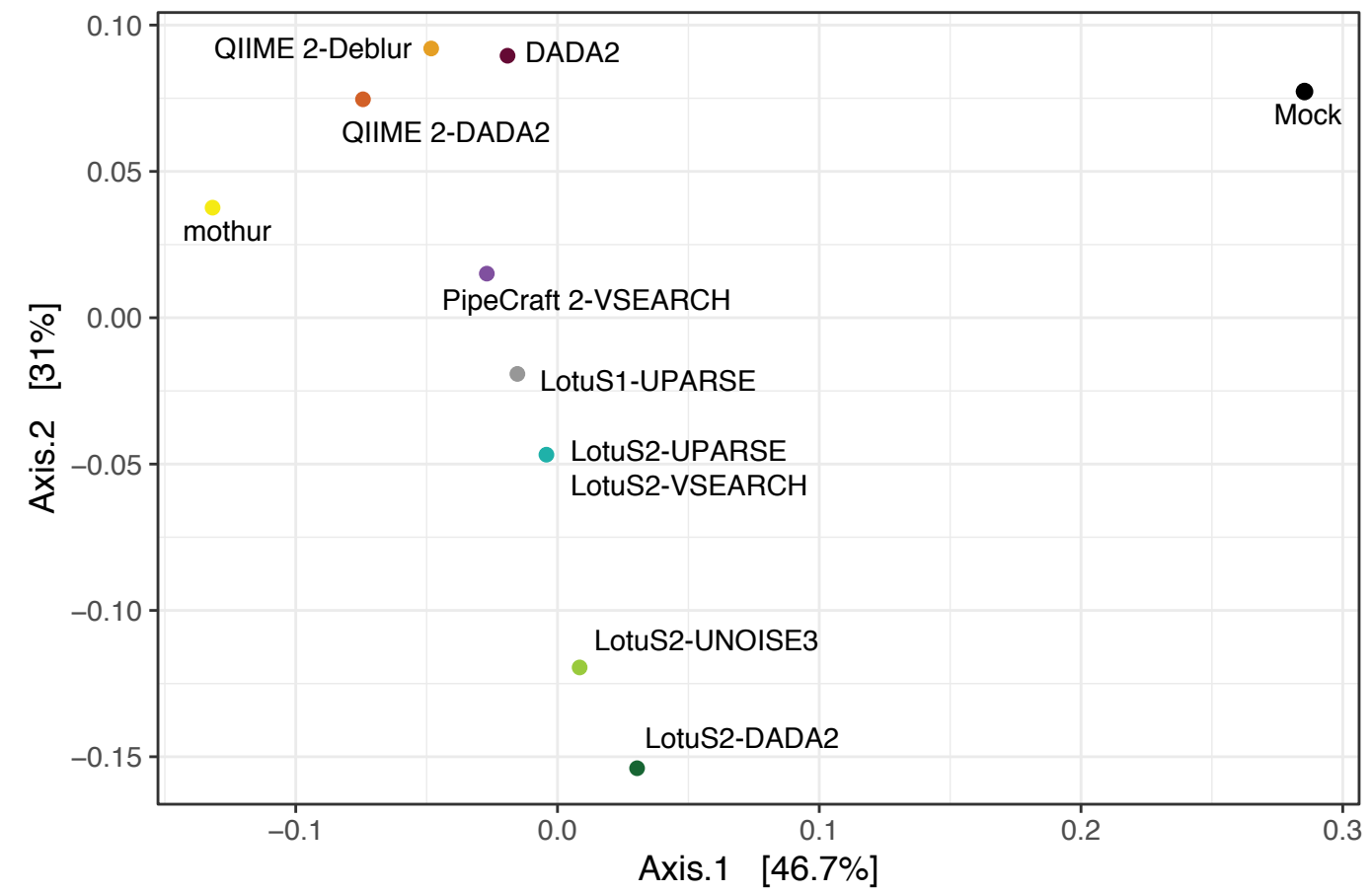

Supplement: Supplementary file 12 — Additional file 11: Supplementary Figure S7. Observed composition of the mock community compared to the composition predicted by each pipeline. A) Relative abundances of the 16 orders having the highest abundance. B) Bray-Curtis distance based PCoA of the observed composition of the mock sample and composition predicted by each pipeline [file 40168_2022_1365_MOESM11_ESM.pdf]

**A**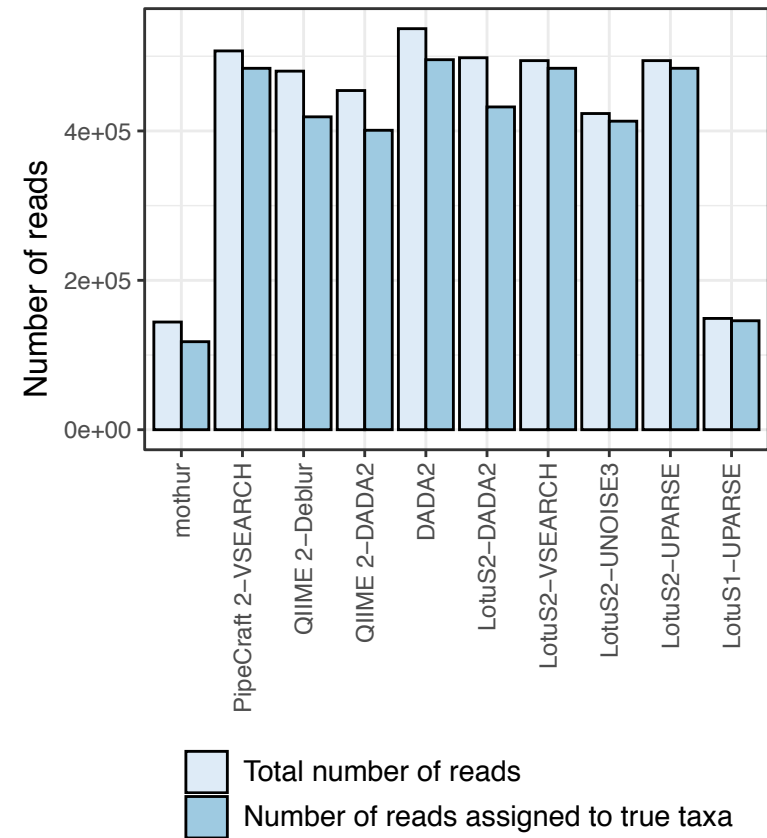**B**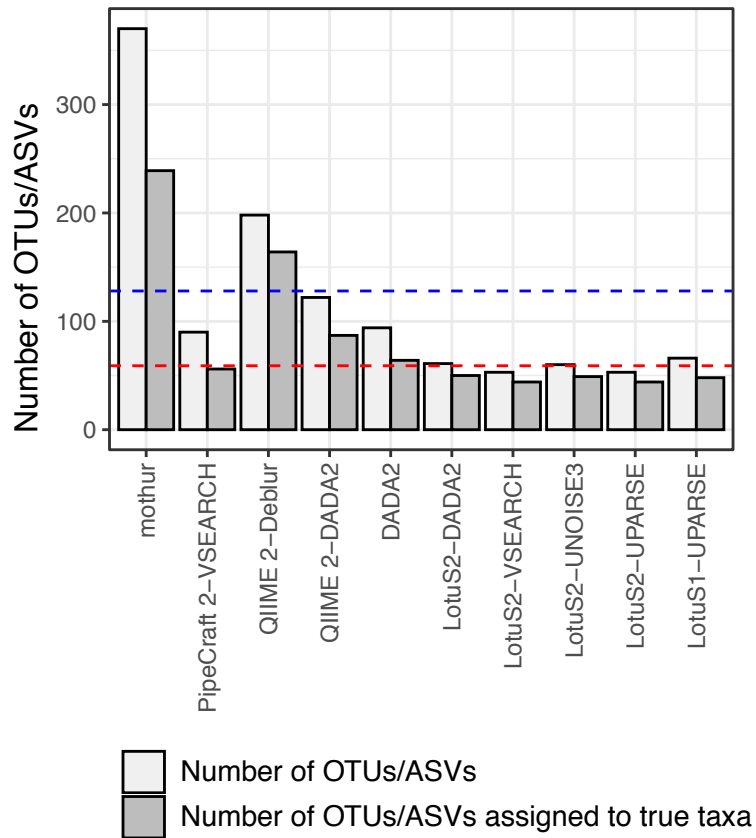

Supplement: Supplementary file 13 — Additional file 12: Supplementary Figure S8. Number of reads and OTUs/ASVs and those assigned true taxa at genus level by each pipeline in the analysis of the mock community. Total number of A) reads retrieved by each pipeline and those assigned to true taxa at genus level B) OTUs/ASVs generated by each pipeline and those assigned to true taxa at genus level. Blue and red line indicates number of 16S gene copies and species, respectively, in the mock community. [file 40168_2022_1365_MOESM12_ESM.pdf]

Fraction of TP reads
  Fraction of TP OTUs/ASVs
  Precision
  Recall
  F-score

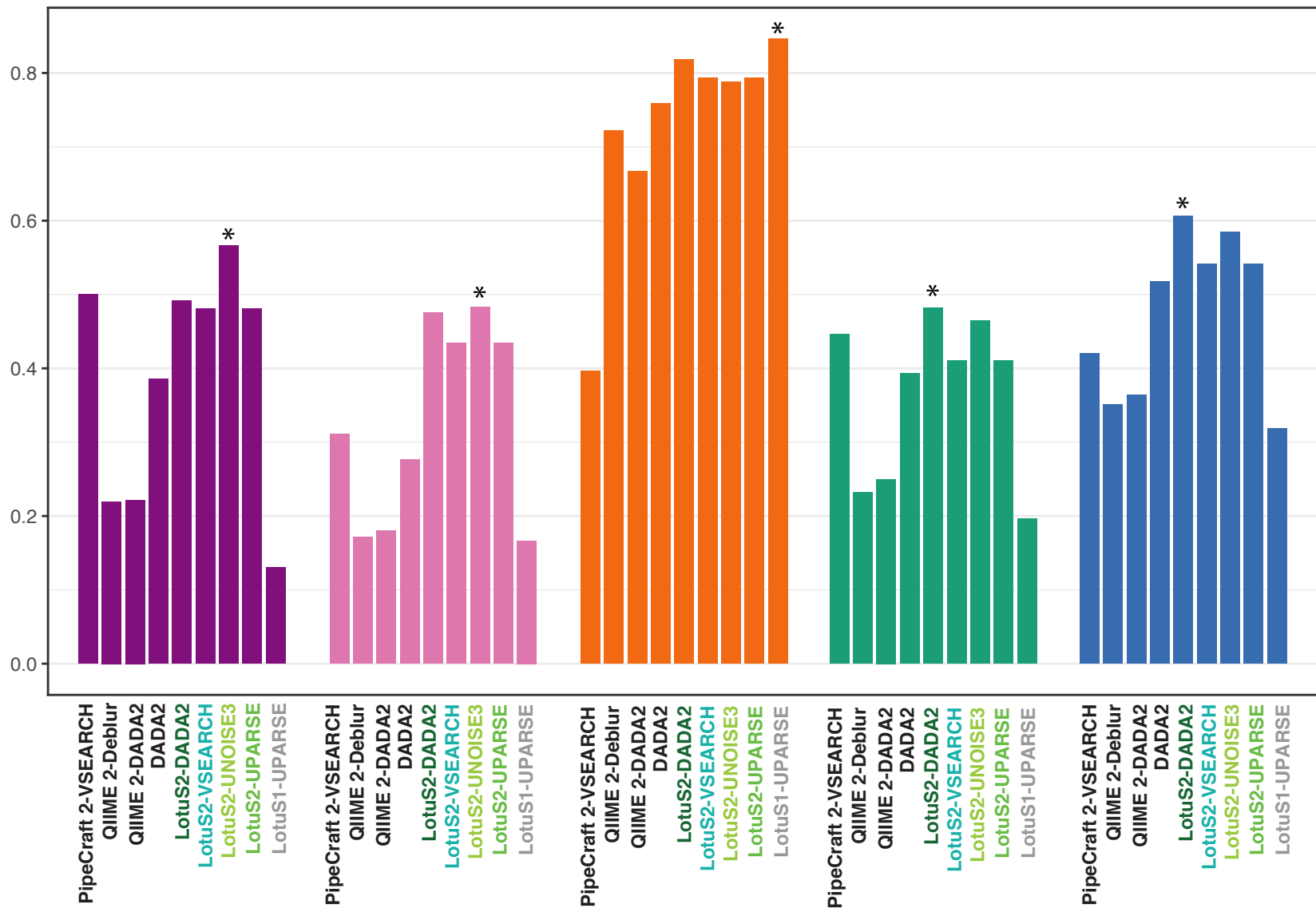

Supplement: Supplementary file 14 — Additional file 13: Supplementary Figure S9. Accuracy of each pipeline in predicting the mock community composition at species level. For benchmarking we compared the fraction of reads assigned to true taxa and both correctly and erroneously recovered taxa at the species level from the mock community. [file 40168_2022_1365_MOESM13_ESM.pdf]
