# Supplementary material for: LotuS2: an ultrafast and highly accurate tool for amplicon sequencing analysis
Source: Microbiome. 2022 Oct 19;10:176. doi: 10.1186/s40168-022-01365-1 (PMC9580208; doi:10.1186/s40168-022-01365-1)
Supplement: Supplementary file 10 — Additional file 9: Supplementary Figure S5. Reproducibility of beta diversity at different read truncation lengths. Reproducibility of sequenced technical replicates by measuring the Bray-Curtis (A and C) and Jaccard distances (B and D) of the microbiome composition among technical replicate samples. Two datasets were used to represent different biomes and amplicon technologies using (A, B) human faecal samples (16S rRNA primer, N=40 replicates) and (C, D) soil samples (16S rRNA, V4-V5 region primers, N=50 replicates). Lower Bray-Curtis or Jaccard distances between technical replicates indicate better reproducibility of community compositions. Default pipeline parameters and recommended settings for each dataset were used (Please see the Supplementary information for further information). [file 40168_2022_1365_MOESM9_ESM.pdf]

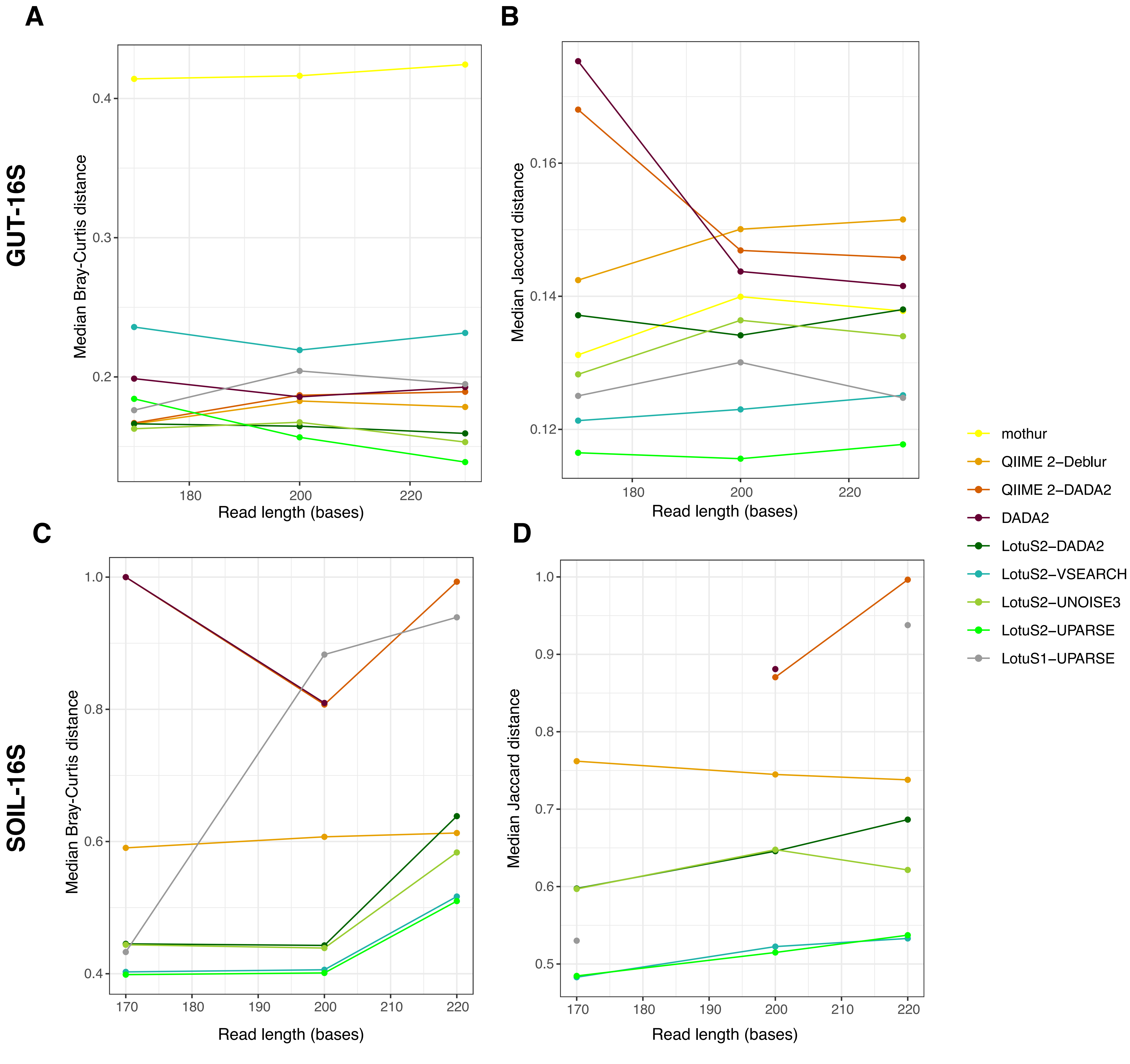

A
B
C
D
GUT-16S
SOIL-16S
Median Bray-Curtis distance
Median Jaccard distance
Read length (bases)
mothur
QIIME 2–Deblur
QIIME 2–DADA2
DADA2
LotuS2–DADA2
LotuS2–VSEARCH
LotuS2–UNOISE3
LotuS2–UPARSE
LotuS1–UPARSE
