# Supplementary material for: LotuS2: an ultrafast and highly accurate tool for amplicon sequencing analysis
Source: Microbiome. 2022 Oct 19;10:176. doi: 10.1186/s40168-022-01365-1 (PMC9580208; doi:10.1186/s40168-022-01365-1)
Supplement: Supplementary file 15 — Additional file 14: Supplementary information. [file 40168_2022_1365_MOESM14_ESM.docx]

**Supplementary Information:**

**Influence of dereplication thresholds, non-default parameters, and read truncation**

Dereplication is the pre-clustering of sequencing reads at 100% nucleotide identity, a commonly used strategy to reduce the computational complexity of sequence clustering [18]. Further, dereplication can be used to filter out sparsely occurring reads that could represent technical artifacts, unlikely to represent true biodiversity. Therefore, LotuS2 uses a “dereplication” filter that can be user defined.

Overall, this filter does not mostly change the number of OTU/ASV counts, with more OTUs/ASVs being recovered when the filter is more relaxed **(Supp. Figure 2A,D,G)**. This is expected because this filter is designed to remove sparse OTUs/ASVs that could both represent technical artefacts as well as extremely rare microbes. However, this did not affect the overall community reproducibility of either gut- or soil-16S samples. However, in soil-ITS samples, we noted a dramatic decrease in BCd between technical replicates at stricter dereplication cut-offs **(Supp. Figure 2H-I)**.

The number of retrieved reads remained very stable independent of filtering stringency; this is expected because the backmapping of mid-quality reads will re-introduce reads not passing the dereplication filter.

LotuS2 uses several default options (-lulu 1, -xtalk 0, -keepUnclassified 0, and -ITSX 1; where “1” means the option is “activated” and “0” means “deactivated”). When activated, -lulu option uses LULU R package [24] to merge OTUs/ASVs based on their co-occurrences; -xtalk option checks for cross-talk [33], -keepUnclassified includes unclassified (i.e. not matching to any taxon in the taxonomy database) OTUs/ASVs in the final matrix, and -ITSx activates the ITSx program [32] to only retain OTUs fitting to ITS1/ITS2 hmm models. The impact of these parameters on the reproducibility of LotuS2 was tested **(Supp. Figure 3).** Overall, non-default options did not change the BCd between the technical replicates except -keepUnclassified 1 notably increasing BCd in gut-16S, while -lulu 0 slightly increased BCd in soil-ITS.

Read length truncation is frequently used to remove the typically low quality 3’ end of reads [8,18]. This is impacting the retrieved read counts as well as observed OTU/ASV diversity. For example, at 170 bp read truncation, mothur, DADA2, and QIIME 2-DADA2 were severely impacted in merging read pairs, failing or only integrating a fraction of read pairs in gut and soil-16S datasets **Supp. Figure 4**). While LotuS2 also had slightly different read and cluster numbers with changing truncation lengths, it was more stable, because reads are merged in the seed extension step after sequence clustering on truncated, high-quality reads are completed **(Supp. Figure 4)**. In shorter or longer read truncations, LotuS2 was still performing the best with the lowest BCd **(Supp. Figure 5A,C)** and Jd **(Supp. Figure 5B,D)** between technical replicates in both gut- and soil-16S datasets.

Taken together, the higher performance of LotuS2 in reproducibility of the dataset was independent of the dereplication parameters and read truncation length.
